# Membrane‐Targeted Nanoparticles for Neuronal Photomodulation

**DOI:** 10.1002/advs.78575

**Published:** 2026-11-01

**Authors:** Ane Escobar, Ana Sánchez‐Iglesias, Davide Bello, Miriam Feijoo Barriga, Alex M. Ascensión, Laura Rodríguez‐Gómez, David Otaegui‐Bichot, Francisco Javier Gil‐Bea, Monica Carril, Marek Grzelczak

**Affiliations:** ^1^ Centro de Física de Materiales (CFM‐MPC), CSIC‐UPV/EHU Donostia‐San Sebastián Spain; ^2^ Instituto Biofisika (CSIC, UPV/EHU) Leioa Spain; ^3^ Fundación Biofísica Bizkaia/Biofisika Bizkaia Fundazioa (FBB) Leioa Spain; ^4^ Neurosciences Area Biodonostia Health Research Institute Donostia ‐ San Sebastián Spain; ^5^ CIBERNED Carlos III Institute Madrid Spain; ^6^ IKERBASQUE Basque Foundation for Science Bilbao Spain; ^7^ Department of Health Sciences Public University of Navarra (UPNA) Pamplona Spain; ^8^ Departamento de Bioquímica y Biología Molecular University of the Basque Country (UPV/EHU) Barrio Sarriena s/n Leioa Spain; ^9^ Donostia International Physics Center (DIPC) Donostia ‐ San Sebastián Spain

**Keywords:** bioorthogonal chemistry, gold bipyramids, neuromodulation, neuroplasmonics, thermoplasmonics

## Abstract

Selective targeting of nanoparticles to the neuronal interface is central for the tailored development of precision neurotechnologies. Here, we report the targeting of gold bipyramids, as a model nanomaterial, on the membrane of primary neurons using a bioorthogonal approach. In a two‐step process, linker molecules anchor to receptors that expose trans‐cyclooctene, which forms a covalent bond with the tetrazine functional group on nanoparticles, leading to the retention of hundreds of nanoparticles per cell. Selective labeling of neuronal membranes with plasmonic nanoparticles via a bioorthogonal strategy enabled inhibition of neuronal activity through photothermal effects under NIR laser irradiation in synchronized cell cultures. The linker and/or nanoparticles alone have a negligible effect on neuromodulation. Nanoparticle retention at the membrane is rapid, requiring only one hour of total incubation, with no cytotoxic effect. These results expand the repertoire of light‐activated nanomaterials for non‐invasive neuromodulation.

## Graphical abstract

Immobilizing gold nanoparticles on the neural interface using a bioorthogonal approach generates localized heat upon exposure to near‐infrared light, temporarily inhibiting primary neuronal activity without damaging the cells. This precise placement and retention of nanoparticles at the cell membrane expand the strategies for non‐invasive neural network modulation.

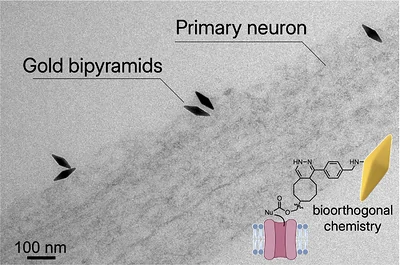

## 1 Introduction

Conventional infrared neural stimulation, based on direct heating of water via pulsed lasers, suffers from relatively high radiant exposures constrained by skin/tissue safety limits and limited spatial resolution (hundreds of micrometers) [1, 2]. Plasmonic nanoparticles offer physical properties—localized surface plasmon resonance (LSPR) converting incoming light into local heat through the Joule effect [3, 4], that can concentrate optical energy down to the nanoscale at relatively low incident power densities. Efficient photothermal actuation requires the excitation wavelength to match the LSPR, which in practice translates to precisely engineering nanoparticle shape and size. In fact, the photothermal effect has been exploited as a therapeutic means not only for cancer treatment via hyperthermia [5, 6, 7], but also for photoregulation of biological activities [8]. The sub‐hyperthermic threshold is particularly relevant for neuronal modulation (stimulation or silencing), where nanoparticles alter neuronal activity by transducing light‐to‐heat [9, 10, 11], as shown in vitro [12, 13, 14, 15, 16, 17, 18, 19], ex vivo [20, 21], or in vivo [22, 23, 24], offering a non‐genetic route to optical neuromodulation, i.e. “neuroplasmonics”. The wireless, genetically free approaches for reversible neuronal silencing may be particularly relevant for conditions involving hyperexcitable circuits, such as drug‐resistant epilepsy or chronic pain.

Neuromodulation is a membrane‐centered phenomenon that requires nanoparticle retention at the cell membrane to maximize the likelihood of converting incoming electromagnetic radiation into heat and then into physiological responses [9, 25, 26]. While selective membrane targeting of molecular transducers is feasible via physical or chemical modifications [27, 28], the immobilization of nanoparticles through molecular recognition is often hindered due to the protein corona [29]. Without a doubt, the bioorthogonal modification of the neuronal membrane via click chemistry is one of the most promising strategies [30], especially when using an intermediate anchor point, a linker that bridges receptors and nanoparticle cargo. As a matter of fact, Ojima et al. [31, 32], have demonstrated high specificity, rapid kinetics, and long‐term retention of molecules to ionotropic glutamate receptors. Despite the promised versatility, validation of their method for materials other than organic dyes remains a pending task.

The present work builds on the approach by Ojima and co‐workers [31], and exploits a bioorthogonal two‐step click‐chemistry method for specific, covalent tethering of gold nanoparticles to neurons. In our approach, the linker containing the AMPAR‐selective antagonist PFQX (6‐pyrrolyl‐7‐trifluoromethyl‐quinoxaline‐2,3‐dione) binds selectively to AMPA‐type glutamate receptors on neurons, exposing a trans‐cyclooctene (TCO) functional group to the bulk medium, which later captures tetrazine (Tz) functionalized nanoparticles by forming a firm covalent bond (Figure 1A,B). The two‐step functionalization process requires 0.5 h of incubation time for both the linker and gold nanoparticles, as verified by confocal fluorescence imaging and electron microscopy, and revealed a several‐fold increase in membrane‐bound nanoparticles after linker pre‐treatment compared to controls. Retention of nanoparticles at the cell membrane via a bioorthogonal approach enhances photothermal modulation upon near‐infrared laser exposure, as assessed by multielectrode array recordings. We found that, regardless of energy dose, the amount of nanoparticles delivered to the membrane controls neuromodulation.

**FIGURE 1:**
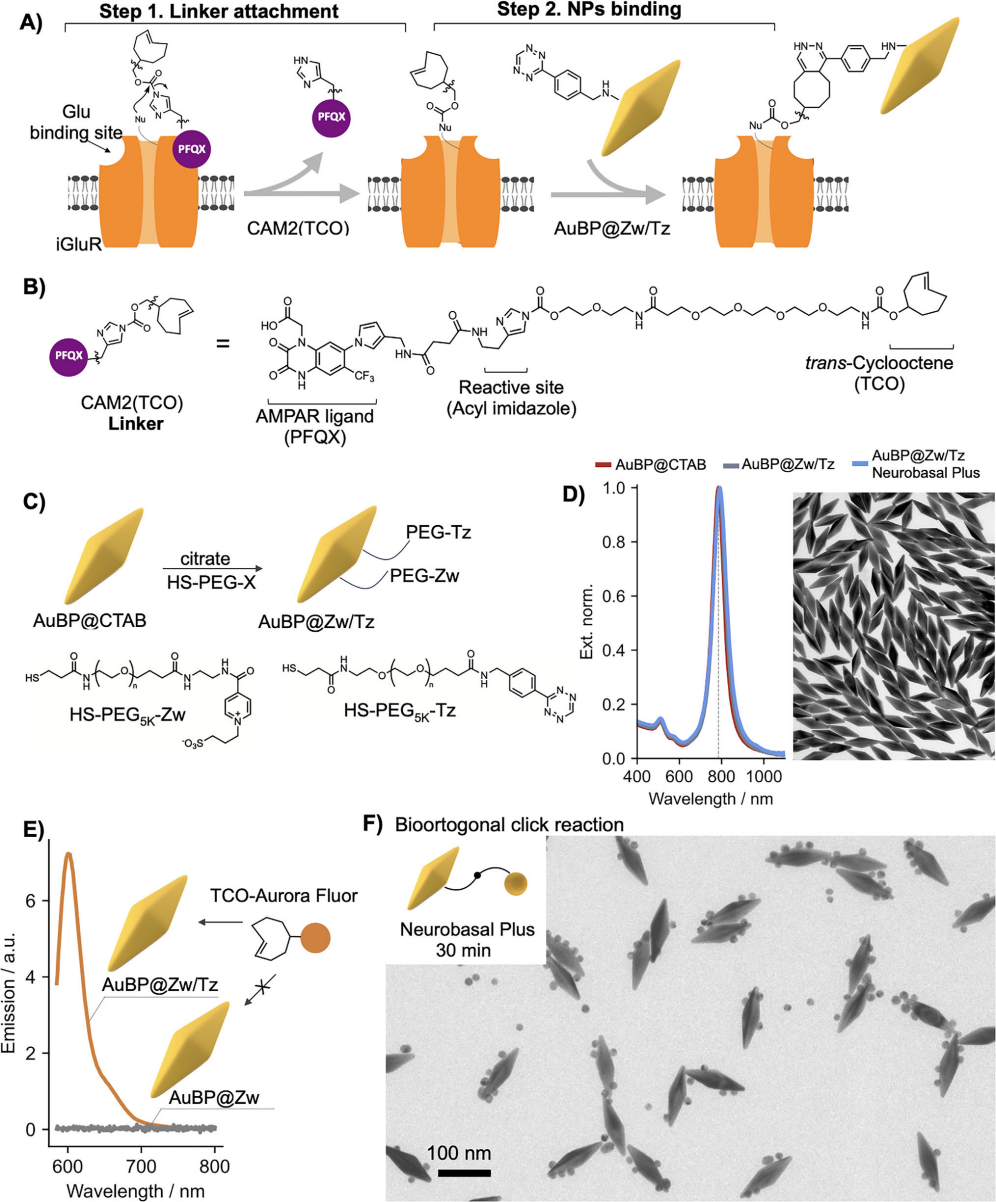
Functionalized gold nanoparticles. (A) Two‐step bioorthogonal immobilization of gold bipyramids (AuBP on the cell membrane. A linker binds to the receptor, followed by a click reaction between the hanging TCO and the tetrazine Tz group on AuBPs. (B) Chemical structures of the linker comprising the AMPAR‐targeting domain, the reactive site, and the TCO terminus. (C) Functionalization of AuBPs with binary surface ligands, HS‐PEG‐Tz and HS‐PEG‐Zw. (D) UV–vis–NIR extinction spectra of AuBPs before and after ligand exchange and stored in Neurobasal Plus. TEM micrograph of AuBPs. (E, F) Control experiments on selective bioorthogonal chemistry in chemically crowded media. E) Binding of an organic dye to the nanoparticle surface, and F) formation of bipyramid‐sphere core‐satellite architecture.

## 2 Results and Discussion

### 2.1 Nanoparticles and Surface Chemistry

We selected gold bipyramids (AuBPs) as a model plasmonic nanomaterial for neuronal stimulation. Because of their sharp tips, AuBPs exhibit exceptionally intense, sharp LSPR in the near‐infrared (NIR) spectral range, as compared to gold nanorods, a common example of anisotropic plasmonic nanoparticles [33, 34]. The narrow linewidth of AuBPs translates into a high absorption cross‐section and thus a photothermal effect [35], which, in practice, reduces the concentration of nanoparticles needed to achieve the desired temperature increase, thereby mitigating potential toxicity and clearance concerns. Also, due to their penta‐folding symmetry and the presence of twin planes, gold bipyramids outperform single‐crystalline gold nanorods in structural stability at elevated temperatures [36, 37], making them promising photothermal agents for use in neuronal photomodulation. We prepared AuBPs according to our recent protocol [38], with dimensions of 82 ± 2 nm in length, 26 ± 1 nm in width, and LSPR centered at 785 nm (Figure 1D).

To enable robust surface chemistry, we first replaced the native surfactant, cetyltrimethylammonium chloride (CTAC), with citrate, then functionalized the surface with binary ligands: HS‐PEG‐Tz, bearing tetrazine, and HS‐PEG‐Zw, zwitterionic functional groups (Section S1.2). Both polymers contained a 5 kDa PEG domain (Figure 1C). The redshift of LSPR by 6 nm indicated the change in local refractive index and thus successful ligand exchange from native surfactant to polymer (Figure 1D). Attempting to functionalize AuBPs with HS‐PEG‐Tz directly from surfactant led to aggregation, presumably due to interactions of the weakly charged Tz group with positive CTAC micelles. The intermediate citrate ligand was needed for efficient anchoring of thiol‐bearing PEG‐Tz [39]. Also, the auxiliary surface ligand with a zwitterionic terminus (Figure 1C) prevented nanoparticles from aggregating in physiological media (Neurobasal Plus), in contrast to nanoparticles stabilized with HS‐PEG‐Tz as the sole surface ligand. Zwitterionic groups are known for their antifouling properties, which are central to the targeted delivery of nanoparticles to the neuronal interface [40, 41, 42]. The AuBPs functionalized with a binary ligand (AuBP@Tz/Zw) remained colloidally stable in Neurobasal Plus for at least 24 h (Figure 1D, Figure S8).

An inverse‐electron‐demand Diels–Alder cycloaddition is the central process in the bioorthogonal chemistry exploited here, in which TCO and Tz termini form covalent bonds, thereby bridging adjacent entities. To demonstrate the chemical specificity of the bioorthogonal method, we hypothesized that organic dyes or spherical nanoparticles can bind to gold bipyramids in a molecularly crowded physiological medium. To evaluate the hypothesis, we incubated AuBPs@Zw/Tz with TCO‐Aurora Fluor 568 in Neurobasal Plus and observed a prominent emission at 600 nm that persisted after three centrifugation cycles (Figure 1E). The non‐specific interactions of the dye with nanoparticles were excluded in a control experiment in which AuBPs functionalized with Zw showed no emission after three cycles of centrifugation (Figure 1E). The bioorthogonal reaction is not limited to molecular labels and can be expanded to nanoparticle systems, resulting in the formation of hybrid structures (Figure 1F). These results confirm the chemical accessibility and reactivity of surface ligands in physiological medium, validating the bioorthogonal approach for targeting nanoparticles at the neuronal interface.

### 2.2 Targeting AuBPs at the Neuronal Interface

We tested the interaction of AuBPs@Zw/Tz with SH‐SY5Y neuroblastoma cells as a model system expressing AMPARs (Figure S12). First, we incubated cells with the linker (5 µM) for 0.5 h. Note that the incubation time of 0.5 h throughout the study was fixed based on previous findings by Ojima et al. [31], who have shown that the AMPAR antagonist—quinoxaline‐2,3‐dione derivative (PFQX)—reaches near‐equilibrium binding within a few minutes in cellular systems. Second, we removed the unbound linker molecules, replenished the cell medium, and incubated the cells with AuBPs@Zw/Tz (0.002 nM in terms of NPs) for 0.5, 1, 3, and 24 h. We removed unbound nanoparticles from the medium (Figure 2A). To visualize gold nanoparticles under a confocal microscope, we functionalized bipyramids with a third surface ligand, HS‐PEG‐Alexa488, which emits at 517 nm (Figure S6). Confocal microscopy images showed weak, diffuse fluorescence in the absence of the linker, i.e., omitting Step 1 (Figure 2A), suggesting limited nanoparticle binding at all time points (Figure 2B and Figure S14, left panel). On the contrary, in the presence of the linker, the emission was pronounced (Figure 2B and Figure S14, right panel). The control experiment, with no nanoparticles, showed no green emission (Figure S15). This analysis qualitatively demonstrates the need for a linker to retain nanoparticles in cell culture.

**FIGURE 2:**
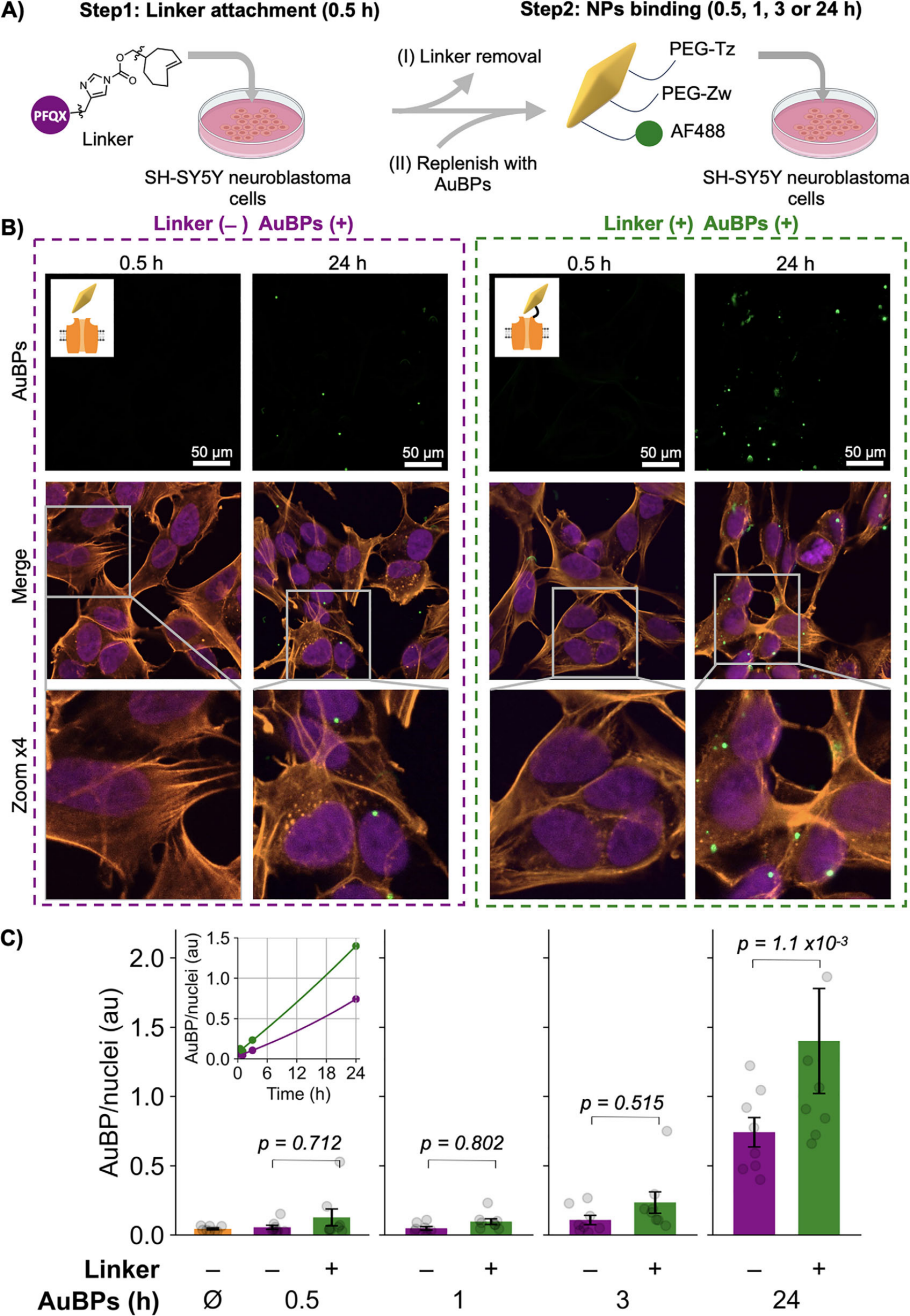
Interaction of AuBPs with SH‐SY5Y cells. (A) Two‐step process for nanoparticles immobilization. AuBPs are functionalized with a ternary, dye‐bearing ligand. (B) Confocal fluorescence images of SH‐SY5Y neuroblastoma cells incubated with AuBPs without a linker (left panel) or with a linker (right panels), for 0.5 or 24 h. color code in merged images: β‐actin (orange), nuclei (purple), AuBPs (green). Figures S16 and S17 show images of 1 and 3 h incubation period and control, respectively. (C) AuBPs/nuclei fluorescence ratio as a function of the presence or absence of linker at given nanoparticle incubation time. Inset: AuBP‐to‐nucleus fluorescence ratio versus incubation time in the presence (green) and absence (violet) of linker. All *p*‐values were obtained using one‐way ANOVA analysis.

Quantitative image analysis of the AuBP‐to‐nucleus fluorescence ratio (Figure 2C) showed no statistically significant differences at early incubation times (0.5–3 h; all *p* > 0.5). In contrast, after 24 h, AuBP‐associated emission increased twofold in the presence of the linker (*p* <0.01). The absence of detectable differences at shorter time points (<3 h) likely reflects the limited spatial resolution of confocal microscopy for resolving individual nanoparticles at the membrane. Importantly, the number of detected AuBPs increases progressively with incubation time (Figure 2C, inset), with a more pronounced effect in linker‐treated neurons.

To further evaluate bioorthogonal chemistry at a short incubation time of 0.5 h, we shifted to linker‐mediated immobilization of nanoparticles on rat primary cortical (CTX) neurons, expressing AMPARs (Figures S14 and S15). We followed a two‐step immobilization workflow (Figure 3A) that comprises incubation of neurons with the linker (0.5 h, 5 µM), its removal, and subsequent incubation with AuBPs@Zw/Tz (0.02 nM) for 0.5 or 24 h. We removed the excess nanoparticles and subjected the cultured cells to transmission electron microscopy (TEM), inductively coupled plasma‐mass spectrometry (ICP‐MS), and cytotoxicity analysis. TEM was used to overcome the spatial‐resolution limits of confocal imaging and definitively distinguish between membrane‐associated and internalized nanoparticles.

**FIGURE 3:**
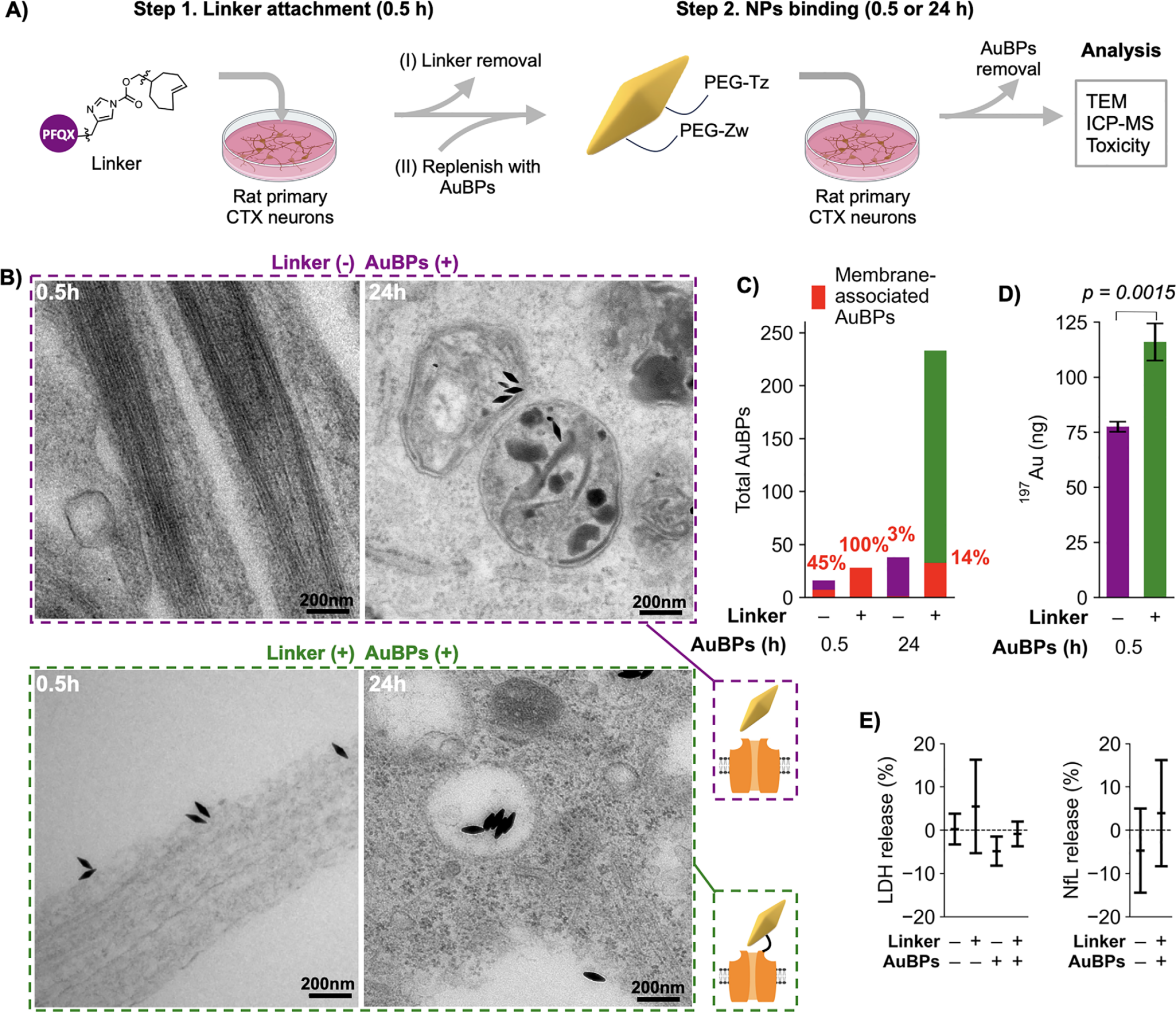
Targeting AuBPs to the membrane of primary neurons. (A) Two‐step, linker‐mediated procedure to immobilize nanoparticles to neurons. The resulting samples were analyzed by TEM, ICP‐Ms and toxicity assays. (B) TEM images of CTX primary neurons incubated with AuBPs either without linker (upper panel) or with linker (lower panel), for 0.5 or 24 h. (C) Quantified number of total AuBPs and membrane‐associated AuBPs (red) obtained from TEM analysis (eight images per condition) for neurons incubated with or without linker for 0.5 or 24 h. (D) ICP‐MS quantification of ^197^Au CTX neuron after 0.5 h incubation with AuBPs in the absence or presence of linker. (E) LDH release (left) and NfL release (right) from neurons treated with linker only, AuBPs only, and mixture of both. Data are shown as percentage release relative to the control baseline. All p‐values were obtained using one‐way ANOVA analysis.

A qualitative TEM inspection showed no AuBPs in the sample at 0.5 h incubation when the linker was excluded (Figure 3B). In contrast, the linker pre‐treatment step led to decoration of the membrane with nanoparticles. At a longer incubation time of 24 h, nanoparticles were found at the membrane and internalized by neurons. We quantified the percentage of nanoparticles retained at the membrane. After a short incubation of 0.5 h, we counted 28 and 16 NPs in samples with and without linker treatment, respectively (Figure 3C; Table S3). Importantly, 100% of nanoparticles were retained at the cell membrane when using the linker. ICP‐MS further confirmed the ratio, showing a 1.5‐fold increase in metallic gold content in linker‐pretreated neurons compared to the condition without linker (*p* = 0.0015) (Figure 3D). These results align with the greater nanoparticle accumulation observed by confocal microscopy after 24 h of incubation in linker‐treated SH‐SY5Y cells. Given the mass of an individual gold bipyramid (∼6 × 10^−^^7^ ng) and the total mass of gold in the sample measured by ICP‐MS and TEM analysis, one obtains the total number of cell‐associated nanoparticles with and without linker treatment, that is, 1.3 × 10^8^ and 2.0 × 10^8^, respectively. Note that while ICP‐MS, by measuring total gold amount, cannot distinguish membrane‐bound from internalized nanoparticles, TEM analysis at the same time point allows this distinction. Given the total number of neurons in a well (∼10^5^), we estimate, on average, the accumulation of ∼650 nanoparticles per cell after 0.5 h incubation. Because individual AuBPs present multiple tetrazine‐bearing PEG chains and are large relative to the spacing between AMPARs within dense postsynaptic clusters, a single particle cannot be assumed to correspond to a single linker or receptor engagement event; we therefore do not derive a linker‐ or receptor‐occupancy estimate from this value, and present it as an approximate measure of linker‐dependent particle accumulation. For a longer nanoparticle incubation time of 24 h, the number of counted nanoparticles located at the membrane remained similar (29) to that after 0.5 h of incubation, but accounted for only 14% of the total bipyramids; 86% clustered in the cytoplasm within the endolysosomal compartment. In the absence of a linker, all counted nanoparticles were clustered in the cytoplasm. Linker pre‐treatment leads to a ten‐fold increase in the number of nanoparticles after 24 h. Our data indicate that retention at the cell membrane depends on the number of active linker molecules and, therefore, on receptor presence.

Finally, the LDH release and neurofilament light chain (NfL) detection analysis indicated membrane integrity and the absence of neuronal injury (Figure 3E). Consistent with these biochemical readouts, qualitative bright‐field inspection of the cultures revealed no overt detachment (Figure S24). Overall, neurons tolerate the two‐step bioorthogonal strategy for nanoparticle attachment.

### 2.3 Photothermal Modulation

To evaluate wireless photothermal neuronal modulation, we cultured CTX primary neurons for 14 days on a Multielectrode Array (MEA) with 16 electrodes, coupled to a continuous‐wave laser at 785 nm with a 0.7 mm beam spot (Figure 4A, Figure S18). Note that photothermal neuromodulation was assessed exclusively in CTX primary neurons rather than SH‐SY5Y cells, since this functional assay requires spontaneous, synchronized network‐level electrical activity, a hallmark of mature and synaptically connected primary neuronal cultures that is not reliably reproduced by immortalized SH‐SY5Y cultures.

**FIGURE 4:**
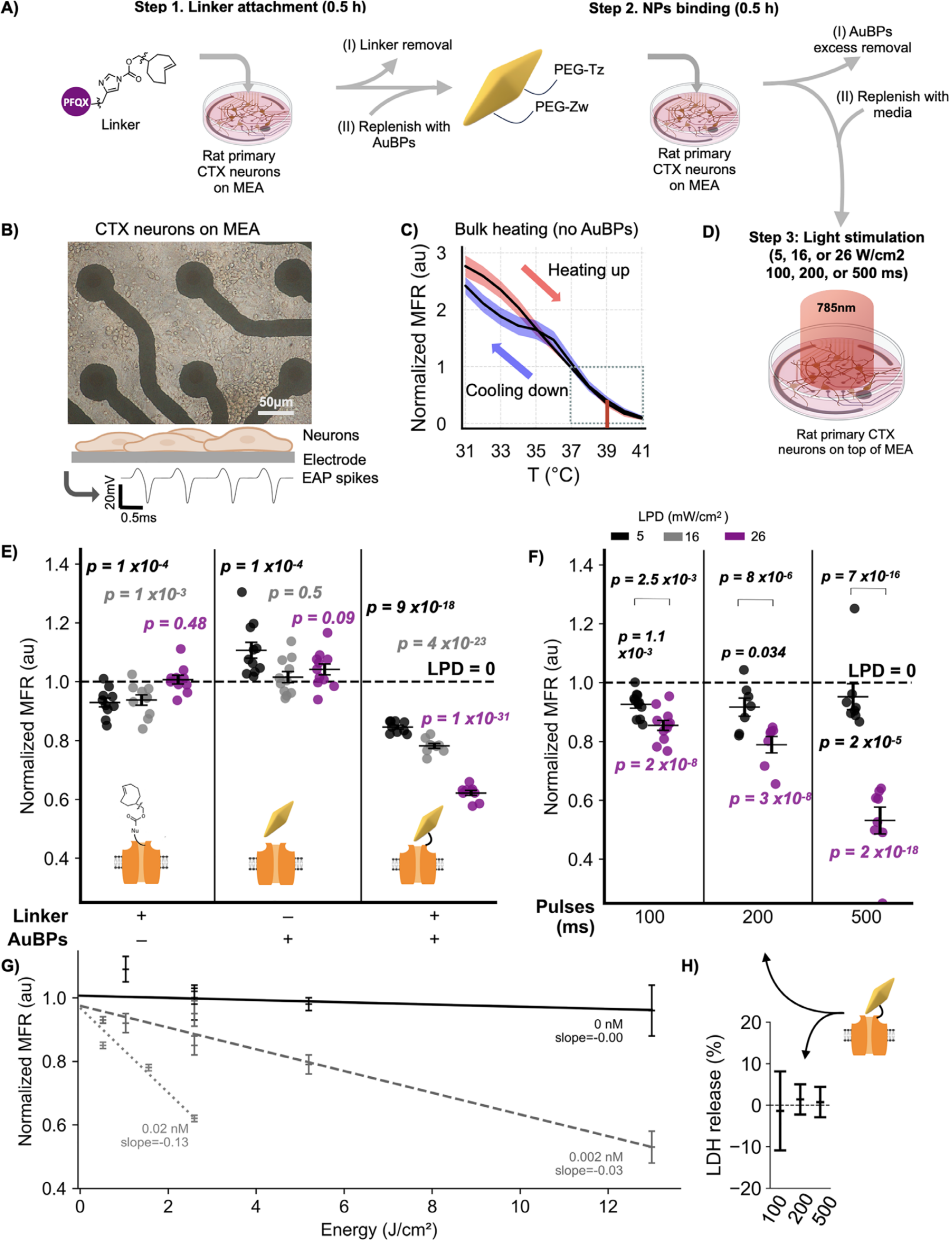
Photothermal modulation of neuronal activity. (A) Two‐step, linker‐mediated targeting of AuBPs to neurons cultured on MEAs. (B) CTX neurons cultured on an MEA and corresponding extracellular action potential (EAP) spike trace. (C) Normalized mean firing rate (MFR) as a function of bulk temperature during a forward scan from 31°C to 41°C and subsequent passive cooling, both in dark conditions. (D) Photothermal stimulation conditions. CTX neurons cultured on MEAs are irradiated with a 785 nm continuous‐wave laser. (E) Normalized MFR after 100 ms laser pulses at laser power densities from 5, 16, to 26 W/cm^2^ in neurons treated with linker only, AuBPs only, and mixture of both. (F) Normalized MFR after laser stimulation with 100, 200, or 500 ms pulse durations at 5 or 26 W/cm^2^ in neurons treated with linker + AuBPs. (G) Normalized MFR as a function of energy dose per pulse for different AuBP concentrations. (H) LDH release after photothermal stimulation with different pulse durations. All values are expressed as mean ± SE, normalized to baseline (dashed black line at MFR = 1, pre‐treatment; LPD = 0) levels, and p‐values are obtained by statistical analysis by one‐way ANOVA. Data in E‐G were obtained from a single MEA well per condition (one neuronal preparation); electrodes were used as the unit of analysis, and p‐values reflect the consistency of the response across the spatial extent of this network.

Coupling the laser to the MEA system had a negligible effect on medium temperature (Figure S19). We also removed all detected artifacts in the MEA signal that arose during the transition from laser states ON/OFF by excluding all recordings within a time window of ±10 ms (Figures S20 and S21).

Before photomodulation, we evaluated neuronal activity under bulk heating in the dark, especially because literature suggests that temperature‐induced activity changes are cell‐dependent [43, 44, 45]. By subjecting the neurons to a forward temperature scan (1°C/min) from 31°C to 41°C, the spontaneous extracellular activity of neurons, measured as the mean firing rate (MFR), progressively decreased, showing a 15% decrease at 41°C relative to their physiological activity reaching ∼14% of its 37°C baseline value (i.e., an ∼86% decrease) at 41°C (Figure 4C and Figure S22). Upon passive cooling (∼0.5°C/min), the neurons recovered MFR to initial levels, indicating that bulk thermal stress is reversible and non‐damaging. The amplitude of extracellular spikes recorded across all electrodes decreased consistently and monotonically as temperature rose above 37°C, reaching a minimum at 41°C (10% loss of spike amplitude), with partial recovery upon cooling (Figure S23). This behavior could arise from two concurrent physical mechanisms. First, the electrical conductivity of aqueous electrolyte solutions increases with temperature due to higher ionic mobility, which reduces the resistance of the extracellular medium and therefore the voltage detected by the MEA electrodes [46]. Second, elevated temperature accelerates the inactivation kinetics of voltage‐gated Na^+^ channels, causing the inactivation gate to close earlier during the depolarisation phase, so that less total Na^+^ enters the cell per spike and the net transmembrane current is reduced [47, 48]. Although other mechanisms could be at play, together, these two mechanisms could act synergistically to attenuate spike amplitude with increasing temperature, and their reversibility upon cooling is consistent with the amplitude recovery upon cooling.

Although cellular processes are typically thermally accelerated within the 31–41°C range studied here, the observed decrease in both MFR and spike amplitude with increasing temperature reflects the suppression of network‐level excitability. The suppression can stem from the combined influence of temperature on membrane conductance, ion channels, and synaptic transmission, reducing the probability of sustained action potential generation [45]. Regardless of the mechanisms ruling the temperature‐induced MFR reduction, our observation sets a thermal baseline, enabling interpretation of neuronal modulation via photothermal effects.

We used a two‐step incubation method to immobilize nanoparticles on neurons cultured on electrodes: first linker (0.5 h, 5 µM), then AuBPs@Zw/Tz (0.5 h, 0.02 nM) (Figure 4A). After each step, we removed the unbound linker or nanoparticles. Upon laser irradiation (100 ms pulse), a reduction in MFR was observed only in the presence of both the linker and the AuBPs. Specifically, as the power density increased from 5 to 16 and 26 W/cm^2^, the MFR dropped by approximately 15%, 30%, and 45%, respectively (Figure 4E). In contrast, neurons treated with either the linker alone or AuBPs alone showed negligible changes in normalized MFR across the same range of power densities (Figure 4E, Figures S25–S28 and S36). The normalized spike amplitude decreased by ∼1% at 26 W/cm^2^, whereas it remained at baseline under control conditions (Figure S33).

Next, we tested laser pulse widths (100, 200, and 500 ms) on MFR at two limiting power densities (5 and 26 W/cm^2^), using a 10‐fold lower concentration of bipyramids (0.002 nM; Figure 4F, Figures S29–S31 and S36–S38). We decreased the nanoparticle concentration to broaden the dynamic range of MFR change. At a lower power density (5 W/cm^2^), pulse duration had little effect on MFR, resulting in a consistent 10% decrease across all pulse durations. At a higher power density (26 W/cm^2^), the MFR decreased to 45% at a pulse duration of 500 ms. In contrast, no significant changes in the MFR were observed for untreated neurons (Figure S32). Our results corroborate observations by Begeng et al. [21], who have demonstrated that millisecond pulses lead to neuronal inhibition. The spike amplitude attenuated ∼10% (Figure S34). Notably, burst rate did not consistently decrease with increasing LPD (Figure S35), in contrast to the clear reduction observed in overall MFR (Figure 4E–G). This dissociation suggests that photothermal stimulation reduces individual neuronal spiking activity without disrupting the network burst structure, that is, without abolishing the synchronized activity that reflects functional connectivity between neurons. Together, the MFR, spike amplitude, and burst rate results indicate that longer pulse durations strengthen the inhibitory photothermal effect by reducing neuronal excitability while preserving the network connectivity. Control neurons, in contrast, remain largely unaffected.

To provide a broader overview of neuronal modulation, we plotted the data as MFR vs. energy dose per pulse (J/cm^2^) for different nanoparticle concentrations. Note that dose per pulse combines power density and pulse duration [49]. The data showed that the slope of MFR vs. energy dose is sensitive to the nanoparticle concentration (Figure 4G and Table S2). Interestingly, a 10‐fold lower nanoparticle concentration yielded a fivefold smaller MFR slope, meaning one needs to deliver a higher energy dose to compensate for the lower nanoparticle number and achieve the same stimulation [50]. The plot in Figure 4G suggests that there is a subtle window of nanoparticle concentration: at low concentration, a mild modulation is invoked across a wider energy dose; at high concentration, an abrupt modulation occurs at a narrow energy dose. Therefore, the results demonstrate that a bioorthogonal approach enables an additional experimental parameter –concentration of gold nanoparticles–, apart from dose per pulse, to wirelessly modulate neuronal activity. Using the concentration‐dependent MFR slope relationship reported above (a 10‐fold reduction in nanoparticle concentration yielding only a fivefold reduction in MFR slope, Figure 4G) and taking the ICP‐MS‐derived reference of ∼650 cell‐associated AuBPs/cell at 0.02 nM (Figure 3C) as an anchor point, we estimate a membrane‐bound density of ∼130 NPs/cell at 0.002 nM, the lowest concentration at which robust photothermal neuromodulation (∼45% MFR suppression) was observed (Figure 4F). This provides an estimated lower bound for functional photothermal neuromodulation under our experimental conditions.

The remaining question concerns the potential mechanism behind the inhibition of neuronal activity. Bulk and photothermal heating consistently reduced MFR, which may indicate temperature‐modulated neuronal activity rather than other mechanisms. For example, reactive oxygen species are less likely here because they require doses above 40 J/cm^2^, nearly double the dose used in the present study. Taking the change in MFR under bulk heating as a reference, where an ∼64% MFR reduction upon a 2°C increase (MFR dropping to ∼36% of its baseline value) from 37 to 39°C, one can postulate that photothermal heating generates a similar 2°C increase at the dose of 2.6 J/cm^2^ ([Au^0^] = 0.02 nM). The reduced spike amplitude provides an additional electrophysiological signature of photothermal heating. Recently, Lee et al. [25], have suggested that reversible neuronal photomodulation requires a mild temperature increase of 2–4°C, eventually transitioning to non‐reversible modulation above 6°C. Since our results demonstrate reversible modulation, we postulate that the temperature increase is within 2–4°C. Of course, the neurons change their behavior as if they were subjected to a temperature rise, but this does not necessarily mean a 2°C temperature increase. Highly localized heat dissipation by gold bipyramids anchored to the membrane can alter ion balance, leading to a macroscopically observable change in MFR without necessarily increasing temperature. Using reliable nanothemometry colocalized with bipyramids will provide further experimental evidence for these mechanisms.

The precision in delivering a relatively small number of nanoparticles at the neuronal interface via a bioorthogonal approach helps mitigate potential phototoxicity arising from the photothermal effect. We observed that LDH release assay values remained within the baseline range (±10%) relative to untreated controls, regardless of pulse duration (Figure 4G), indicating the absence of membrane damage. Consistent with the unchanged NfL release, quantitative assessment of neurite/axonal integrity and qualitative bright‐field inspection of the cultures after linker, nanoparticle incubation, and laser stimulation revealed no detachment or neurite fragmentation (Figure S24). Moreover, the post‐stimulation recordings in our system showed no evidence of sustained activity suppression following the stimulation conditions tested (Figure S39).

While it might be counterintuitive that thermal energy invariably increases neuronal firing, our observation of a photothermal inhibition aligns with the thermodynamic properties of the neuronal membrane under sustained heating. One possible explanation is that, within the temporal regime of the NIR pulses used here (100–500 ms), thermal equilibration of membrane proteins could enhance the temperature‐dependent activity of the electrogenic Na+/K+ ATPase pump (Q10 ≈ 3), potentially contributing to membrane hyperpolarization [45]. Localized heating may also alter the kinetics of voltage‐gated sodium channel inactivation and membrane resistance, which could reduce neuronal excitability and attenuate the propagation of excitatory inputs [51]. This interpretation is consistent with established biophysical evidence that transient heating reversibly alters neuronal membrane electrical properties, including membrane capacitance [52], an effect later formalized in a quantitative intramembrane mechanoelectrical framework showing that mild, transient temperature increases reversibly perturb membrane electrical properties without inducing permanent structural damage [53]. These considerations provide possible biophysical explanations for the reduced MFR and spike amplitude observed here, although the present extracellular recordings do not allow us to distinguish between these mechanisms or establish a specific molecular basis for the inhibition. This mechanism may differ from the use of ultra‐short (microsecond) laser pulses typically employed to evoke transient, depolarizing capacitive currents for neural activation [20, 52]. Thus, under the specific spatio‐temporal heating conditions investigated here, the observed response is consistent with reversible neuronal inhibition rather than excitation, while the underlying cellular mechanisms remain to be established.

## 3 Conclusions

We demonstrated that gold bipyramids, a model plasmonic system with precisely designed surface chemistry comprising binary ligands and bearing both zwitterionic and tetrazine groups, can be retained orthogonally at the neuronal interface by first attaching a molecular linker to AMPA receptors. The combination of precision nanochemistry (high‐quality, colloidally stable nanocrystals in physiological media) and a bioorthogonal approach (selective surface ligands and linker) showed that the two‐step immobilization strategy can retain hundreds of nanoparticles per cell with 1 h of total incubation time. We tested the labeled neurons in wireless photothermal neuromodulation using an NIR laser at a moderate energy dose (13 J/cm^2^ per pulse), showing a decrease in MFR and in the extracellular spike waveform, consistent with reversible bulk heating of the primary neurons. Our approach provides precision in neuromodulation by not only controlling the number of photoactive nanoparticles at the neuronal interface but also their spatial distribution (membrane vs. cytoplasm) within a given time window. This precision can be central to resolving the mechanisms of neuronal photomodulation, which involve considerable complexity across a vast parameter space, including laser wavelength, power density, pulse width, nanoparticle optical properties, concentration, spatial distribution in cells, and even maintaining colloidal stability in physiological medium during neuromodulation [54, 55]. The proposed method for targeting nanoparticles at the neural interface is certainly a step forward and will allow for further simplification of parameter space to elucidate, in further work, the physiological origins of photothermal modulation, e.g., disentanglement of receptor‐triggered and membrane capacitance pathways [52, 56].

Looking forward, the linker‐mediated bioorthogonal approach is material‐agnostic, allowing anchoring of other nanoparticle types, including magnetic nanocrystals and quantum dots. Notably, functional photothermal neuromodulation was achieved even under conditions of comparatively low, non‐saturating linker‐receptor engagement, rather than requiring near‐complete receptor occupancy. We view this as a favorable feature for eventual in vivo translation: following systemic or local delivery, effective linker and nanoparticle concentrations reaching target neurons are expected to be substantially lower than those used here in static cell culture, owing to the blood‐brain barrier, cerebrospinal fluid clearance, interstitial diffusion, and off‐target competition. The chemistry can also be further customized to enable linker molecules to bind specifically to various ion channels or neurotransmitter receptors (e.g., GABAergic, dopaminergic, or voltage‐gated channels). In doing so, one could temporally channel the photothermal effect to distinct cellular populations, broadening the translational scope of this platform and enabling applications in diverse disease models. Within the AMPAR family itself, subtype‐selective targeting of calcium‐permeable AMPARs, which lack GluA2 and have been implicated in excitotoxic neuronal vulnerability across several neurological disorders [57], could similarly offer improved cellular specificity over the pan‐AMPAR engagement achieved with the PFQX‐based linker used here. Finally, while the short incubation time (0.5 h) favors predominantly membrane‐associated nanoparticles, the long‐term intracellular fate of internalized, non‐degradable AuBPs remains an open question for future studies using longer‐term exposure paradigms.

## Author Contributions

**Ane Escobar**: Conceptualization, Methodology, Investigation, Formal analysis, Funding acquisition, Project administration, Data curation, Writing – original draft, Writing – review & editing. **Ana Sánchez‐Iglesias**: Methodology, Investigation, Formal analysis, Data curation, Writing – review & editing. **Davide Bello**: Methodology, Investigation, Writing – review & editing. **Miriam Feijoo Barriga**: Methodology, Investigation. **Alex M. Ascensión**: Formal analysis. **Laura Rodríguez‐Gómez**: Methodology. **David Otaegui Bichot**: Methodology, Validation, Funding acquisition. **Francisco Javier Gil‐Bea**: Methodology, Validation, Funding acquisition, Writing – review & editing. **Mónica Carril**: Conceptualization, Methodology, Validation, Funding acquisition, Writing – review & editing. **Marek Grzelczak**: Conceptualization, Supervision, Funding acquisition, Project administration, Data curation, Writing – original draft, Writing – review & editing.

## Conflicts of Interest

The authors declare no conflicts of interest.

## Supporting information




**Supporting File**: advs78575‐sup‐0001‐SuppMat.docx.

## Data Availability

The data that support the findings of this study are available on request from the corresponding author. The data are not publicly available due to privacy or ethical restrictions.
