# Supplementary material for: Membrane‐Targeted Nanoparticles for Neuronal Photomodulation
Source: Adv Sci (Weinh). 2026 Nov 1:e78575. Online ahead of print. doi: 10.1002/advs.78575 (PMC13723740; doi:10.1002/advs.78575)
Supplement: Supplementary file 1 [file ADVS-9999-e78575-s001.docx]

**Membrane-targeted nanoparticles for neuronal photo-modulation**

**Supporting Information**

[*Ane Escobar*](mailto:marek.g@csic.es)*^1^, Ana Sánchez-Iglesias^1^, Davide Bello^2,3^, Miriam Feijoo Barriga^2,3^, Alex Martínez-Ascensión^4^, Laura Rodríguez-Gómez^4^, David Otaegui-Bichot^4,5^, Francisco Javier Gil-Bea^4,5,6,7^, Mónica Carril^2,6,8^, Marek Grzelczak^1,9,*^*

^1^ Centro de Física de Materiales (CSIC-UPV/EHU), Paseo Manuel de Lardizabal 5, 20018 Donostia-San Sebastián, Spain

^2^ Instituto Biofisika, Barrio Sarriena, S/N, 48940 Leioa, Spain

^3^ Fundación Biofísica Bizkaia/Biofisika Bizkaia Fundazioa (FBB), Leioa E-48940, Spain

^4^ Neurosciences Area, Biodonostia Health Research Institute, 20014, San Sebastián, Spain.

^5^ CIBERNED, Carlos III Institute, 28031, Madrid, Spain.

^6^ IKERBASQUE, Basque Foundation for Science, 48013, Bilbao, Spain.

^7^ Department of Health Sciences, Public University of Navarra (UPNA), Pamplona, Spain.

^8^ Departamento de Bioquímica y Biología Molecular, University of the Basque Country (UPV/EHU) Barrio Sarriena s/n, 48940, Leioa, Spain

^9^ Donostia International Physics Center (DIPC), Paseo Manuel de Lardizabal 4, 20018 Donostia-Sebastián, Spain

*Corresponding author. Email: [marek.g@csic.es](mailto:marek.g@csic.es)

Table of Contents

[**1. Materials and methods**	4](#_Toc241476885)

[**1.1. Synthesis and functionalization of gold bipyramids**	4](#_Toc241476886)

[**Chemicals**	4](#_Toc241476887)

[**Instrumentation**	4](#_Toc241476888)

[**Synthesis of gold seeds**	4](#_Toc241476889)

[**Synthesis of gold bipyramids**	5](#_Toc241476890)

[**Functionalization of gold bipyramids with citrate**	6](#_Toc241476891)

[**Functionalization of gold bipyramids with binary surface ligands (Tetrazine and zwitterion groups) - AuBPs@PEG-Zw/Tz**	6](#_Toc241476892)

[**Colloidal stability of AuBPs@PEG-Tz/Zw in physiological conditions**	7](#_Toc241476893)

[**Control experiments demonstrating Tz-TCO chemistry in physiological medium**	7](#_Toc241476894)

[**Functionalization of gold bipyramids ternary surface ligands (AuBPs@PEG-Zw/Tz/Alexa488)**	9](#_Toc241476895)

[**Antifouling effect of zwitterion groups**	10](#_Toc241476896)

[**1.2. Synthesis of ligands**	11](#_Toc241476897)

[**General information**	12](#_Toc241476898)

[**Synthesis of HS-PEG5K-Tz (5)**	13](#_Toc241476899)

[***tert*-Butyl (4-(1,2,4,5-tetrazin-3-yl)benzyl)carbamate (1)**	13](#_Toc241476900)

[**(4-(1,2,4,5-Tetrazin-3-yl)phenyl)methanaminium chloride (2)**	14](#_Toc241476901)

[**Trityl-S-PEG5K-Tz (4)**	14](#_Toc241476902)

[**HS-PEG5K-Tz (5)**	14](#_Toc241476903)

[**Synthesis of HS-PEG-3K-AlexaFluor-488 (9)**	15](#_Toc241476904)

[**TrtS-PEG-3K-AlexaFluor-488 (8)**	16](#_Toc241476905)

[**HS-PEG-3K-AlexaFluor-488 (9)**	16](#_Toc241476906)

[**Synthesis of CAM2-TCO (10)**	16](#_Toc241476907)

[**Synthesis of HS-PEG5K-ZW (18)**	17](#_Toc241476908)

[***tert*-Butyl (2-(isonicotinamido)ethyl)carbamate (13)**	17](#_Toc241476909)

[**3-(4-((2-((*tert*-Butoxycarbonyl)amino)ethyl)carbamoyl)pyridin-1-ium-1-yl)propane-1-sulfonate (15)**	17](#_Toc241476910)

[**3-(4-((2-Ammonioethyl)carbamoyl)pyridin-1-ium-1-yl)propane-1-sulfonate trifluoroacetate (16)**	18](#_Toc241476911)

[**Trt-S-PEG5K-ZW (17)**	18](#_Toc241476912)

[**HS-PEG5K-ZW (18)**	19](#_Toc241476913)

[**1.3. In vitro experiments**	19](#_Toc241476914)

[**Primary cortical neurons culture**	19](#_Toc241476915)

[**SH-SY5Y neuroblastoma cells culture**	20](#_Toc241476916)

[**Assessment of AMPAR expression**	20](#_Toc241476917)

[**Pharmacological validation of AMPAR functional expression**	22](#_Toc241476918)

[**Attachment of AuBPs to cell membrane**	23](#_Toc241476919)

[**Confocal imaging**	23](#_Toc241476920)

[**Transmission electron microscopy**	23](#_Toc241476921)

[**Inductively Coupled Plasma – Mass Spectrometry**	24](#_Toc241476922)

[**Laser-MEAs setup for photothermal neuromodulation and recording**	24](#_Toc241476923)

[**Temperature monitoring during pulsed laser irradiation**	24](#_Toc241476924)

[**Neuronal network activity modulation**	25](#_Toc241476925)

[**Table S2**. Number of electrodes analysed per condition in Figure 4E-G.	26](#_Toc241476926)

[**Temperature experiment in MEAs with CTX Neurons**	27](#_Toc241476927)

[**Computational Analysis**	27](#_Toc241476928)

[**Cytotoxicity and phototoxicity evaluation**	28](#_Toc241476929)

[**Statistical analysis**	28](#_Toc241476930)

[**2. Results and discussion**	28](#_Toc241476931)

[**Comment on the antifouling effect of zwitterion groups**	28](#_Toc241476932)

[**Comment on pharmacological validation of functional AMPAR in primary CTX neurons**	29](#_Toc241476933)

[**Comment on thermal suppression of neuronal activities**	35](#_Toc241476934)

[**References**	51](#_Toc241476935)

# **1. Materials and methods**

## **1.1. Synthesis and functionalization of gold bipyramids**

### **Chemicals**

Gold (III) chloride trihydrate (HAuCl_4_, ≥ 99 %), hexadecyltrimethylammonium chloride (CTAC, 25 wt % in water), citric acid (≥ 99.5 %), sodium borohydride (NaBH_4_), hexadecyltrimethylammonium bromide (CTAB, ≥ 99 %), silver nitrate (AgNO_3_, ≥ 99 %), 8-hydroxinoline (8-HQL, ≥ 98.5 %), poly (sodium 4-styrenesulfonate) (Na-PSS, Mw~70000 g/mol) and sodium citrate tribasic dihydrate (≥ 99 %) were purchased from Merck. Mercapto poly (ethylene glycol) carboxylic acid (SH-PEG-COOH, Mw: 5 Kg/mol) was purchased from Rapp Polymere. Ethanol (99.9 %) was purchased from Scharlab. Gibco Neurobasal^®^ Medium was purchased from Fisher Scientific. Aurora™ Fluor 568 TCO was purchased from Cambridge Research Biochemicals. All chemicals were used without further purification. Milli-Q water (resistivity 18.2 MΩ·cm at 25 °C) was used in all experiments. All glassware and magnetic stirrer bars were washed with aqua regia, rinsed with Milli-Q water, and dried before use.

### **Instrumentation**

Optical extinction spectra were recorded using an Agilent Cary 3500 UV-visible spectrophotometer. Emission spectra were recorded using an Agilent Cary Eclipse Fluorescence spectrometer. TEM images were acquired with a FEI Tecnai G2 20 TWIN transmission electron microscope operating at an acceleration voltage of 200 kV. Hydrodynamic diameter and zeta potential measurements were determined using a Zetasizer Ultra Malvern. To determine the average hydrodynamic diameter, 10 measurements were taken in each medium (water and Neurobasal Plus). To determine the average zeta potential, 5 measurements were taken in each medium.

### **Synthesis of gold seeds**

The gold seeds were prepared according to the protocol by A. Sánchez-Iglesias et al. ^[1]^ by fast reduction of gold precursor with NaBH_4_ in the presence of a cationic surfactant (CTAC) and citric acid in a 20 mL scintillation vial. To an aqueous CTAC solution (10 mL, 50 mM) was added HAuCl_4_ (0.05 mL, 50 mM) followed by stirring for ~3 min until complexation of gold precursor with surfactant (transparent, yellowish solution). Next, citric acid (0.05 mL, 1 M) was added followed by the injection of freshly prepared NaBH_4_ (0.25 mL, 25 mM) under vigorous stirring at 20 °C. The solution turned from yellow to brownish immediately. After 2 min, the seed solution was placed in a silicone oil bath and heated at 80 ºC for 90 min under stirring. The solution colour turned gradually from brown to red, indicating an increase in nanoparticle size. Finally, the thermally treated seed solution was removed from the oil bath, stored at room temperature, and used without further treatment. The final concentration of metallic gold was 0.25 mM.

### **Synthesis of gold bipyramids**

Gold bipyramids were synthesized according to the protocol by A. Sánchez-Iglesias et al.^[1]^ The growth solution was prepared by dissolving 182.2 mg of CTAB in an aqueous CTAC solution (100 mL, 100 mM) in a water bath at 35 ºC. The mixture was stirred for 10 min. After dissolution of the binary surfactant mixture, HAuCl_4_ solution (1 mL, 25 mM) was added and the solution was stirred at 35 ºC for 5 min to obtain a transparent yellowish solution. Subsequently, AgNO_3_ (0.3 mL, 10 mM) and 8-HQL (1 mL, 0.4 M in ethanol) were added, and the color of the growth solution changed from yellowish to colorless. After 30 seconds, gold seed solution (1.25 mL, 0.25 mM) was added to the growth solution under vigorous stirring. After 15 min, an aliquot of 8-HQL (0.75 mL, 0.4 M in ethanol) was added dropwise, and the mixture was then kept for 30 min in a water bath at 35 °C under stirring. Finally, the gold bipyramids dispersion was centrifuged twice (30 min, 3900 RCF) to remove excess reagents and redispersed with 10 mM CTAB to a final metallic gold concentration of 0.5 mM. The dimensions of the gold bipyramids were determined by TEM image analysis, measuring more than 200 randomly selected nanoparticles. The final dimensions of gold bipyramids were 82 ± 2 nm (length) and 26 ± 1 nm (width).

**Figure S1.** Representative TEM images of gold bipyramids at different magnifications.

**Figure S2.** Size distribution of gold bipyramids.

### **Functionalization of gold bipyramids with citrate**

The as-prepared gold bipyramids were functionalized with citrate according to the previous protocol ^[2]^. A suspension of gold bipyramids (50 mL, 0.5 mM) in CTAB aqueous solution (10 mM) was subjected to three cycles of centrifugation (30 min, 3900 RCF) and redispersion with Na-PSS (50 mL, 0.15 % wt) to remove the CTAB from the nanoparticle surface. PSS-stabilized gold bipyramids were centrifuged (30 min, 3900 RCF), redispersed in sodium citrate (50 mL, 5 mM), and incubated overnight. Finally, the gold bipyramids were subjected to two additional centrifugations (30 min, 3900 RCF) and redispersion cycles using sodium citrate (50 mL, 5 mM), yielding stable dispersions of citrate-stabilized gold bipyramids. The final concentrations of metallic gold and sodium citrate were 0.5 mM and 2.2 mM, respectively.

### **Functionalization of gold bipyramids with binary surface ligands (Tetrazine and zwitterion groups) - AuBPs@PEG-Zw/Tz**

Mercapto poly (ethylene glycol) tetrazine (SH-PEG-Tz, Mw: 5 Kg/mol) and mercapto poly (ethylene glycol) zwitterion (SH-PEG-Zw, Mw: 5 Kg/mol) were used as target surface ligands (see below for detailed information on the synthesis). The ligand exchange procedure was followed as described in the previous report ^[3]^. An aqueous solution of SH-PEG-Tz (0.4 mL) containing 5 molecules per nm^2^ of nanoparticle surface (calculated from TEM and UV-Vis measurements) was added dropwise to the citrate-stabilized gold bipyramids solution (20 mL, 0.5 mM) under stirring at 25 ºC. After 10 min, an aqueous solution of SH-PEG-Zw (0.25 mL) containing 25 molecules per nm^2^ of nanoparticle surface was added dropwise to the nanoparticle dispersion under stirring. The mixture was allowed to react for 1h at 25 ºC. PEG-modified gold bipyramids (AuBPs@PEG-Zw/Tz) were centrifuged twice (40 min, 3900 RCF) and dispersed in water. The final concentration of metallic gold was 3.6 mM.

### **Colloidal stability of AuBPs@PEG-Tz/Zw in physiological conditions**

The solution containing AuBPs@PEG-Tz/Zw (22 µL, 3.6 mM) was diluted into Gibco Neurobasal^®^ medium (2 mL) in a quartz cuvette (optical path=1 cm), and the extinction spectra were monitored for 24 h.

### **Control experiments demonstrating Tz-TCO chemistry in physiological medium**

To demonstrate that tetrazine (Tz) groups are available on the surface of gold bipyramids in physiological medium, we have conducted two control experiments:

1) Anchoring organic dye to the nanoparticle through the Tz-TCO covalent bonds;

To target the nanoparticle surface with a dye, we used Aurora™ Fluor 568 TCO, which emits light at 601 nm (**Figure S3**).

**Figure S3.** Emission and absorption spectra of an aqueous dye solution, Aurora™ Fluor 568 TCO.

An aqueous solution of Aurora™ Fluor 568 TCO containing 5 molecules per nm^2^ of nanoparticle surface was added to a dispersion of AuBPs@PEG-Tz/Zw (2 mL, 0.5 mM) in Neurobasal medium under stirring at 25 ºC. The mixture was allowed to react for 30 min. After three cycles of centrifugation (40 min, 3900 RCF), nanoparticles were dispersed in water. Finally, we measured the emission of the nanoparticles targeted with the dye, determining a band located at 601 nm, characteristic of the Aurora™ Fluor 568 dye. The emission spectrum was obtained using an excitation wavelength of 578 nm.

2) Anchoring 5 nm spherical nanoparticles to AuBPs@PEG-Tz/Zw through click chemistry.

Citrate-stabilized spherical gold nanoparticles with a diameter of 5.3 ± 0.4 nm (Figure S4) were synthesized following the protocol developed by Jordi Piella et al.^[4]^ The surface functionalization was performed by adding mercapto poly (ethylene glycol) trans-cyclooctene (6 mg, 0.5 mL, Mw: 3 Kg/mol) to the as-prepared spherical gold nanoparticles (20 mL, 0.27 mM) under stirring at room temperature. The mixture was allowed to react for 1h, and the gold nanospheres were centrifuged twice (40 min, 20600 RCF) and finally dispersed in water. The final metallic gold concentration was 2.5 mM.

**Figure S4.** Spherical gold nanoparticles used to anchor to AuBPs through bioorthogonal chemistry: a) Representative TEM image and b) size distribution.

The Tz-compatible gold nanoparticles (33 µL, 2.5 mM) were added dropwise to the solution containing AuBPs@PEG-Tz/Zw (0.5 mL, 0.5 mM) in Neurobasal medium under vortex mixing. The binary mixture was allowed to react for 30 min, and the colloidal solution was centrifuged twice (30 min, 3900 RCF) and finally dispersed in water. The final core-satellite structure was characterized by TEM, demonstrating the selectivity of click chemistry in physiological medium.

**Figure S5.** Representative TEM images of core-satellite structure comprising AuBPs@PEG-Zw/Tz core and spherical nanoparticles satellite obtained through the covelent bond formation and physiological medium.

### **Functionalization of gold bipyramids ternary surface ligands (AuBPs@PEG-Zw/Tz/Alexa488)**

Mercapto poly (ethylene glycol) Alexa 488 (SH-PEG-Alexa488, Mw: 5 Kg/mol) mercapto poly (ethylene glycol) tetrazine (SH-PEG-Tz, Mw: 5 Kg/mol), and mercapto poly (ethylene glycol) zwitterion (SH-PEG-Zw, Mw: 5 Kg/mol) were used as target polymer ligands. An aqueous solution of SH-PEG-Alexa488 (0.25 mL) containing 5 molecules per nm^2^ of nanoparticle surface was added dropwise to gold bipyramids solution (20 mL, 0.5 mM) under stirring at 25 ºC. After 10 min, an aqueous solution of SH-PEG-Tz (0.4 mL) containing 5 molecules per nm^2^ of nanoparticle surface was added dropwise to the mixture under stirring. After 10 min, an aqueous solution of SH-PEG-Zw (0.25 mL) containing 25 molecules per nm^2^ of nanoparticle surface was added dropwise to the mixture under stirring. The mixture was allowed to react for 1h at 25 ºC. PEGylated gold bipyramids were centrifuged three times (40 min, 3900 RCF), with the last cycle dispersing the nanoparticles in water. The final metallic concentration was 4.7 mM. The emission of AuBPs@PEG-Zw/Tz/Alexa488 was located at 517 nm using an excitation wavelength of 494 nm.

**Figure S6.** Emission and absorption spectra of an aqueous solution of SH-PEG-Alexa488.

**Figure S7.** Emission spectrum of AuBPs@PEG-Zw/Tz/Alexa488 in aqueous solution featuring an emission band characteristic of Alexa dye.

### **Antifouling effect of zwitterion groups**

To demonstrate the antifouling effect of the zwitterion groups, we measured the hydrodynamic diameter of 30 nm gold nanospheres stabilized with PEG-COOH and with PEG-Tz/PEG-Zw in both water and Neurobasal Plus medium. We synthesized citrate-stabilized spherical gold nanoparticles (30 nm in diameter) following the protocol developed by Jordi Piella et al.^[4]^ Then, spherical gold nanoparticles were functionalized with mercapto poly (ethylene glycol) carboxylic acid (SH-PEG-COOH, Mw: 5 Kg/mol) by adding an aqueous solution of SH- PEG-COOH (1.4 mg, 0.5 mL) to the as-prepared spherical gold nanoparticles (7 mL, 0.48 mM) under stirring at 25 ºC. The mixture was allowed to react for 1 h, and the gold nanospheres were centrifuged twice (40 min, 8780 RCF) and finally dispersed in water. Citrate-stabilized spherical gold nanoparticles were functionalized with mercapto poly (ethylene glycol) tetrazine (SH-PEG-Tz, Mw: 5 Kg/mol) and mercapto poly (ethylene glycol) zwitterion (SH-PEG-Zw, Mw: 5 Kg/mol) by adding dropwise an aqueous solution of SH-PEG-Tz (0.2 mL) containing 5 molecules per nm^2^ of nanoparticle surface to the as prepared spherical gold nanoparticles (7 mL, 0.48 mM) under stirring at 25 ºC. After 10 min, an aqueous solution of SH-PEG-Zw (1.4 mg, 0.5 mL) was added dropwise to the nanoparticle solution under stirring. The mixture was allowed to react for 1h. PEG-modified gold nanospheres (Au30nm@PEG-Tz/PEG-Zw) were centrifuged twice (40 min, 8780 RCF) and finally dispersed in water. The final metallic gold concentration of both samples was 3 mM. Samples for determining the hydrodynamic diameter and zeta potential were prepared by diluting 50 µL of Au30nm@PEG in 2 mL of water or in 2 mL of Neurobasal plus medium.

**Figure S8.** A) Hydrodynamic diameter of 30 nm gold nanospheres functionalized with PEG-COOH and with PEG-Zw/PEG-Tz in water, and after 24 h of incubation in Neurobasal plus. B) Zeta potential values of 30 nm gold nanospheres functionalized with PEG5K-COOH and with PEG5K-Zw/PEG5K-Tz in water, and after 24 h of incubation in Neurobasal plus.

## **1.2. Synthesis of ligands**

Surface ligands carry a thiol groups on one end and either Tz or Zwitterion functional groups on the opposite end. The AMPAR linker carries in one terminus the AMPAR ligand PFQX and on the other terminus the bioorthogonal “click” moiety TCO.

### **General information**

Air and moisture-sensitive reactions were carried out under an atmosphere of nitrogen gas pre-dried through a Drierite® column and in oven-dried glassware. Room temperature (rt) refers to 18-25 °C. All evaporation and concentration steps were performed under reduced pressure using Büchi Labortechnik rotary evaporators. All chemicals (from Sigma Aldrich, Fisher Scientific and Fluorochem) were of synthetic grade and were used without further purification. Anhydrous solvents were purchased and used by cannulating under nitrogen atmosphere. Polyethylene glycol polymers were purchased from Rapp-Polymere (Tübingen, Germany). Alexa Fluor™ 488 NHS Ester was purchased from ThermoFisher Scientific.

The course of reactions was followed by thin-layer chromatography (TLC) using aluminium plates coated with silica gel (Silica gel 60 F254 Merck). TLC plates were examined under UV light (254 nm and 366 nm) before being visualised with anisaldehyde-sulfuric acid, alkaline potassium permanganate or ceric sulfate-sulfuric acid and developed by heating.

Flash column chromatography was carried out on an automated purification system (Büchi Pure), using silica gel Büchi Ecoflex cartridges and binary gradients of dichloromethane, methanol, ethyl acetate, hexane, and acetone. Polyethylene glycol polymers (PEGs) were purified by size exclusion chromatography using PD-10 desalting columns (containing 8.3 mL of Sephadex® G25 resin, GE HealthCare, 8.3 mL).

NMR spectra were recorded on a Bruker Avance II 300 or 500 MHz instrument. 1H spectra were obtained using deuterated chloroform (CDCl_3_) or other indicated solvents. Chemical shifts were reported in ppm (parts per million) relative to the signal of the chosen locking solvent. Coupling constants are given in Hertz (Hz). The abbreviations for the multiplicity of the proton, carbon and fluorine signals are as follows: singlet (s), broad singles (br s), doublet (d), triplet (t), quartet (q), and multiplet (m).

Analytical, semi-preparative and preparative high-performance liquid chromatography (HPLC) analyses and purifications were performed on a ThermoFisher Vanquish HPLC system operating in tandem with a Orbitrap Exploris MX, which was employed to determine the accurate mass of eluting peaks. Elution was carried out using binary gradients of water and acetonitrile containing 0.1% formic acid, or 10 mM ammonium acetate in water and acetonitrile, using C18 a Hypersil C18 250x4.6 mm analytical column, or either a Hypersil C18 250x20 mm or a Hypersil C18 250x30 mm preparative columns.

Residual water on isolated compounds was removed by freeze-drying on a Christ Alpha 3-4 LCS-basic apparatus.

### **Synthesis of HS-PEG5K-Tz (5)**

**Figure S9.** Synthetic route for the preparation of HS-PEG5K-Tz (5), including tetrazine functionalization and final deprotection to yield the free thiol.

### ***tert*-Butyl (4-(1,2,4,5-tetrazin-3-yl)benzyl)carbamate (1)**

Compound **1** was synthesized using a modified version of the procedure previously reported by Yang and coworkers.^[5]^ *tert*-Butyl (4-cyanobenzyl)carbamate (500 mg, 2.15 mmol, 1.0 equiv), formamidine acetate (2.3 g, 21.5 mmol, 10.0 equiv) and zinc triflate (393 mg, 1.1 mmol, 0.5 equiv) were loaded in a heavy walled pressure tube containing a magnetic stirring bar. *N,N’*-dimethylformamide (3 mL) was added, followed by hydrazine monohydrate (5.2 mL, 107.5 mmol, 50 equiv). The tube was flushed with nitrogen, sealed and warmed to 70 ºC with stirring for 6 hours. After cooling to room temperature, the mixture was poured into a large beaker flask, 10 mL of water was added and the resulting mixture was cooled to 0 ºC with an ice bath. Acetic acid was slowly added until pH 3, then a solution of sodium nitrite (2.97 g, 43.0 mmol, 20.0 equiv) dissolved in 10 mL of water was slowly added under vigorous stirring, and a bright pink precipitate formed. The mixture was allowed to slowly warm to room temperature under stirring, then dichloromethane (60 mL) was added, the layers separated and the aqueous layer extracted with dichloromethane (3 x 20 mL). The combined organic layers were washed with brine, dried over sodium sulfate, filtered and concentrated under reduced pressure to furnish a bright pink solid material. Purification on a Büchi Pure automated system using a 12g Büchi Ecoflex silica cartridge and an isocratic gradient of dichloromethane and acetone (30 mL/min, 5% acetone, rt = 26 min) furnished compound **1** as a bright pink solid (258 mg, 42% yield). ^1^H NMR (300 MHz, Chloroform-*d*) *δ* 10.21 (s, 1H), 8.60 (d, *J* = 8.3 Hz, 2H), 7.52 (d, *J* = 8.2 Hz, 2H), 4.98 (s, 1H), 4.45 (d, *J* = 6.1 Hz, 2H), 1.48 (s, 9H).

### **(4-(1,2,4,5-Tetrazin-3-yl)phenyl)methanaminium chloride (2)**

Compound **1** (60 mg, 0.21 mmol, 1.0 equiv) was dissolved in dry dichloromethane (2 mL) under an atmosphere of nitrogen. A solution of dry hydrochloric acid dioxane (4M, 418 μL, 1.67 mmol, 8.0 equiv) was added using a Hamilton® microsyringe at room temperature, and the resulting mixture was stirred for 16 hours. TLC analysis confirmed complete conversion, and the mixture was concentrated under reduced pressure, triturated with cold diethyl ether (twice), and dried under high vacuum, funishing hydrochloride **2** (49 mg) in quantitative yield. ^1^H NMR (300 MHz, Methanol-*d4*) *δ* 10.38 (s, 1H), 8.84 – 8.50 (m, 2H), 7.75 (d, *J* = 8.2 Hz, 2H), 4.28 (s, 2H). These data are in good agreement with the literature values.^[6]^

### **Trityl-S-PEG5K-Tz (4)**

α-NHS Ester-ω-Tritylthio PEG 5000 Da **3** (Rapp Polymere cat. No. 135000-41-35, 290 mg, 54.6 μmol, 1.0 equiv) was loaded in round bottom flask under an atmosphere of nitrogen. Dry dichloromethane (5 mL) was added, followed by compound **2** (36.6 mg, 163.8 μmol, 3.0 equiv) and *N,N’*-diisopropylethylamine (49 μL, 273 μmol, 5.0 equiv). The flask was sealed and the resulting pink mixture gently stirred for 16 hours. The mixture was then diluted with dichloromethane (10 mL), washed with saturated aqueous ammonium chloride (4 x 10 mL), brine (2 x 10 mL), the combined aqueous layers were extracted with dichloromethane (3 x 5 mL), and the combined organics were dried onto sodium sulfate, filtered and concentrated under reduced pressure to give a pink solid. This material was dissolved in milliQ water (1 mL) and loaded on a PD-10 desalting column previously flushed with 50 mL of milliQ water. Elution was carried out with milliQ water, collecting 1 mL fractions. These were freeze-dried individually and each tube was analysed by 1H-NMR (using CDCl_3_ as an NMR solvent) prior to pooling. The desired pure compound was present in tubes from 3 to 7, thus the pink solid material in each tube was re-dissolved with dichloromethane, tubes 3-7 combined and concentrated to furnish 270 mg (92% yield) of compound **4**. ^1^H NMR (300 MHz, Chloroform-*d*) *δ* 10.21 (s, 1H, Tz-*H*), 8.57 (d, J = 8.3 Hz, 2H), 7.55 (d, J = 8.3 Hz, 2H), 7.45 – 7.35 (m, 8H), 7.32 – 7.17 (m, 13H), 7.05 – 6.96 (m, 1H), 6.17 – 6.07 (m, 1H), 4.54 (d, J = 6.0 Hz, 2H), 3.91 – 3.83 (m, 3H), 3.80 – 3.54 (m, 596H – polyethylene glycol chain), 3.54 – 3.44 (m, 1H), 3.44 – 3.33 (m, 5H), 2.48 (t, J = 7.4 Hz, 2H), 2.41 (t, J = 6.9 Hz, 2H), 2.05 (t, J = 7.5 Hz, 2H), 2.00 – 1.89 (m, 2H). ^13^C NMR (75 MHz, CDCl_3_) *δ* 173.3, 171.0, 166.3, 157.8, 144.8, 144.8, 130.5, 129.6, 128.8, 128., 127.95, 126.7, 95.1, 77.4, 70.6 (polyethylene glycol chain), 70.6, 70.4, 70.3, 70.0, 69.9, 69.7, 46.1, 43.1, 39.3, 35.4, 33.1, 27.8, 25.7.

### **HS-PEG5K-Tz (5)**

Compound **4** (270 mg, 50 μmol, 1.0 equiv) was dissolved in dichloromethane (8 mL), the mixture cooled to 0 ºC using an ice bath, and triisopropylsilane (514 μL, 2.5 mmol, 50.0 equiv) was added, followed by trifluoroacetic acid (384 μL, 5.0 mmol, 100.0 equiv). As soon as the trifluoroacetic acid was added, the pink mixture turned to an intense yellow colour, indicating the formation of the trityl radical. The colour quickly returned to bright pink, confirming the reduction of the free trityl radical to the colourless triphenylmethane by-product by the silane reagent. The procedure was repeated one more time, then the mixture was allowed to warm to room temperature and the stirring continued for 30 min. The mixture was then concentrated under reduced pressure, the oily pink residue triturated twice with cold hexane and sonicated to aid separation from the silane residues, the solvent was carefully removed each time by aspiration with a glass pipette, taking care not to remove the pink solid. The pink precipitate was then triturate twice more with cold diethyl ether, then dried under high vacuum to furnish compound **5** (246 mg, 96% yield). The compound was stored in a glass vial flushed with nitrogen prior to sealing and kept at - 20 ºC to minimise dimerisation of the free thiol. ^1^H NMR (300 MHz, Chloroform-*d*) *δ* 10.21 (s, 1H, Tz-*H*), 8.57 (d, *J* = 8.3 Hz, 2H), 7.55 (d, *J* = 8.2 Hz, 2H), 6.96 – 6.80 (m, 1H), 4.55 (d, *J* = 6.1 Hz, 2H), 4.50 – 4.43 (m, 2H), 3.86 (dd, *J* = 5.8, 4.0 Hz, 7H), 3.81 – 3.33 (m, 1165H, polyethylene glycol chain), 2.96 (t, *J* = 7.1 Hz, 2H), 2.61 (t, *J* = 7.1 Hz, 2H), 2.50 – 2.38 (m, 2H), 1.99 – 1.84 (m, 2H). ^13^C NMR (75 MHz, CDCl_3_) *δ* 173.3, 171.0, 166.3, 157.8, 144.8, 144.8, 130.5, 129.6, 128.8, 128., 127.95, 126.7, 95.1, 77.4, 70.6 (polyethylene glycol chain), 77.2, 95.1, 128.5, 128.8, 129.4, 157.8, 166.2, 171.0, 173.1.

### **Synthesis of HS-PEG-3K-AlexaFluor-488 (9)**

**Figure S10.** Synthesis of HS-PEG-3K-AlexaFluor-488 (9), showing conjugation of the fluorophore to PEG and subsequent deprotection to obtain the thiol-functionalised product.

### **TrtS-PEG-3K-AlexaFluor-488 (8)**

ɑ-Amino-ω-tritylthio PEG 5000 Da **7** (Rapp Polymere cat. No. 135000-20-41, 37.7 mg, 7.8 μmol, 1.0 equiv) was dissolved in 1.3 mL of a 10 mM, pH 8.3 aqueous solution of sodium hydrogen carbonate. The resulting mixture was added to a screw cap microcentrifuge tube containing Alexa Fluor™ 488 succinimidyl ester **6** (5 mg, 7.8 g μmol, 1.0 equiv). The tube was sealed, wrapped in aluminium foil and shaken for 2 days at room temperature. The mixture was loaded on a PD-10 desalting column previously flushed with 50 mL of milliQ water. Elution was carried out with milliQ water, collecting 1 mL fractions. These were freeze-dried individually and each tube was analysed by 1H-NMR (using CDCl_3_ as an NMR solvent) prior to pooling. The desired pure compound was present in tubes from 2 and 3, thus the solid material in each tube was re-dissolved with dichloromethane, tubes 2-3 combined and concentrated to furnish 43.3 mg (quantitative yield) of compound **8**.

### **HS-PEG-3K-AlexaFluor-488 (9)**

Compound **8** (45.3 mg, 8.4 μmol, 1.0 equiv) was dissolved in dichloromethane (2 mL), the mixture cooled to 0 ºC using an ice bath, and triisopropylsilane (91 μL, 0.42 mmol, 50.0 equiv) was added, followed by trifluoroacetic acid (64.5 μL, 0.84 mmol, 100.0 equiv). The procedure was repeated one more time, then the mixture was allowed to warm to room temperature and the stirring continued for 30 min. The mixture was then concentrated under reduced pressure, the oily dark green residue triturated twice with cold hexane and sonicated to aid separation from the silane residues, the solvent was carefully removed each time by aspiration with a glass pipette, taking care not to remove the solid material. The precipitate was then triturate twice more with cold diethyl ether, then dried under high vacuum to furnish compound **9** (41 mg, 95% yield). The compound was stored in a glass vial flushed with nitrogen prior to sealing and kept at - 20 ºC to minimize dimerization of the free thiol.

### **Synthesis of CAM2-TCO (10)**

**Figure S11**. Chemical structure of CAM2-TCO (10).

Compound **CAM2-TCO (10)** was prepared according to the procedure previously reported by Wakayama ^[7]^ and with some of the modifications detailed in the work of Fiala *et al.*^[8]^ The final purification was carried out by HPLC chromatography using a gradient of 10 mM ammonium acetate and acetonitrile (rt = 44.6 min, linear gradient from 5% to 35% acetonitrile in 60 min, Hypersil C18 250x30 mm column, 18 mL/min eluant flow). *m/z* (ES+) Found: (M+H)^+^ 1106.4616. C_50_H_67_F_3_N_9_O_16_ requires *M*^+^, 1106.4652

### **Synthesis of HS-PEG5K-ZW (18)**

**Figure S12.** Synthetic pathway for HS-PEG5K-ZW (18), detailing intermediate formation, PEG coupling, and final deprotection to yield the zwitterionic thiol-functionalized polymer.

### ***tert*-Butyl (2-(isonicotinamido)ethyl)carbamate (13)**

Isonicotinoyl chloride hydrochloride (1.1 g, 6.3 mmol, 1.0 equiv) was suspended in dry dichloromethane (15 mL) in a round bottom flask under and atmosphere of nitrogen. Catalytic dimethylaminopyridine (15 mg, 0.13 mmol, 0.02 equiv) was added, followed by *N,N-*diethylisopropylamine (3.3 mL, 18.9 mmol, 3.0 equiv). The suspension cleared up and *N*-Boc-ethylenediamine (1.2 mL, 7.55 mmol, 1.2 equivalent) was then added at room temperature, and the resulting mixture stirred for 16 hours. The mixture was then washed with a saturated aqueous solution of ammonium chloride (3 x 10 mL), saturated sodium hydrogen carbonate (2 x 10 mL), brine (2 x 10 mL), dried onto sodium sulfate, filtered and concentrated under reduced pressure. The resulting crude material was purified on a Büchi Pure automated system using a 40g Büchi Ecoflex silica cartridge and an isocratic gradient of dichloromethane and methanol (45 mL/min, rt = 2.5 min at 5% methanol) furnishing compound **13** as an off-white solid (2.5 g, 67% yield). ^1^H NMR (300 MHz, D_2_O) δ 8.89 (d, *J* = 6.4 Hz, 1H), 8.25 (d, *J* = 6.1 Hz, 1H), 3.48 (t, *J* = 5.7 Hz, 1H), 3.28 (t, *J* = 5.7 Hz, 1H), 1.30 (s, 5H). ^13^C NMR (75 MHz, D_2_O) δ 164.9, 158.7, 149.3, 143.2, 125.6, 81.0, 40.8, 39.5, 28.0.

### **3-(4-((2-((*tert*-Butoxycarbonyl)amino)ethyl)carbamoyl)pyridin-1-ium-1-yl)propane-1-sulfonate (15)**

Intermediate **13** (390 mg, 1.5 mmol, 1.0 equiv) was dissolved in dry methanol (5 mL), under an atmosphere of nitrogen. 1,3-propanesultone **14** (179.5 mg, 1.5 mmol, 1.0 equiv) dissolved in dry methanol (2 mL) was added and the resulting mixture stirred at room temperature for 16 hours. The mixture was then concentrated under reduced pressure, the residue redissolved in a 50:50 mixture of water and acetonitrile and purified on a Büchi Pure automated system using a 12g Ecoflex C18 reverse phase cartridge (30 mL/min, rt = 6.0 min at 40% acetonitrile) furnishing compound **15** as a faint yellow waxy solid (427 mg, 75% yield). ^1^H NMR (300 MHz, D_2_O) δ 9.01 (d, *J* = 6.3 Hz, 1H), 8.27 (d, *J* = 6.1 Hz, 1H), 4.79 (t, *J* = 7.4 Hz, 1H), 3.48 (t, *J* = 5.6 Hz, 1H), 3.27 (t, *J* = 5.8 Hz, 1H), 2.93 (t, *J* = 7.2 Hz, 1H), 2.43 (p, *J* = 7.4 Hz, 1H), 1.30 (s, 4H). ^13^C NMR (75 MHz, D_2_O) δ 164.9, 158.2, 149.2, 145.7, 126.3, 80.8, 60.2, 47.0, 40.4, 39.0, 29.6, 27.6, 26.1.

### **3-(4-((2-Ammonioethyl)carbamoyl)pyridin-1-ium-1-yl)propane-1-sulfonate trifluoroacetate (16)**

Compound **15** (194 mg, 0.5 mmol, 1.0 equiv) was loaded in a round bottom flask together with a magnetic stirting bar; the flask was then flushed with nitrogen and capped with a rubber seal. A needle connected to a bubbler was introduced to provide a vent, and neat trifluoroacetic acid (3 mL) was added dropwise with stirring. The material fully dissolved in the trifluoroacetic acid, and the resulting mixture was stirred for 3 hours at room temperature. The mixture was then places under high vacuum using a cold trap in order to remove the trifluoroacetic acid, thus furnishing the title compound as trifluoroacetate salt (sticky solid, 198 mg, quantitative yield).^1^H NMR (300 MHz, MeOD) δ 9.10 (d, *J* = 6.2 Hz, 2H), 8.36 (d, *J* = 6.1 Hz, 2H), 4.80 (m, 2H), 3.65 (t, *J* = 5.7 Hz, 2H), 3.14 (t, *J* = 5.8 Hz, 2H), 2.79 (t, *J* = 6.8 Hz, 2H), 2.38 (p, *J* = 7.0 Hz, 2H). ^13^C NMR (75 MHz, MeOD) δ 163.7, 148.7, 145.8, 126., 60.1, 46.9, 39.1, 37.6, 26.8.

### **Trt-S-PEG5K-ZW (17)**

Compound **16** (30.2 mg, 75.3 μmol, 4.0 equiv) and α-NHS ester-ω-tritylthio PEG 5000 Da **3** (Rapp Polymere cat. No. 135000-41-35, 100 mg, 18.8 μmol, 1.0 equiv) were loaded in round bottom flask under an atmosphere of nitrogen. Dry *N,N’*-dimethylformamide (0.5 mL) was added, followed by *N,N’*-diisopropylethylamine (33 μL, 188.3 μmol, 10.0 equiv). The resulting mixture was stirred for 16 hours, then concentrated under reduced pressure and the residue re-dissolved in milliQ water (1 mL). The resulting solution was loaded on a PD-10 desalting column previously flushed with 50 mL of milliQ water. Elution was carried out with milliQ water, collecting 1 mL fractions. These were freeze-dried individually and each tube was analysed by 1H-NMR (using CDCl_3_ as an NMR solvent) prior to pooling. The desired pure compound was present in tubes from 3 to 6, thus the off-white solid material in each tube was re-dissolved with dichloromethane, tubes 3-6 combined and concentrated to furnish 99 mg (96% yield) of compound **17**

^1^H NMR (300 MHz, MeOD) δ 9.25 (s, 2H), 8.47 (d, *J* = 20.9 Hz, 2H), 7.52 – 7.06 (m, 6H), 4.93 (s, 3H), 3.65 (m, 219H - ^1^H NMR (300 MHz, MeOD) δ 9.24 (dd, *J* = 9.7, 6.5 Hz, 1H), 8.50 (d, *J* = 6.4 Hz, 0H), 3.65 (s, 74H), 2.89 (td, *J* = 6.9, 2.8 Hz, 1H), 2.62 – 2.44 (m, 1H), 2.30 (t, *J* = 7.4 Hz, 0H), 1.85 (p, *J* = 6.6 Hz, 0H), 1.44 – 1.25 (m, 1H).), 2.90 (s, 2H), 2.70 (s, 1H), 2.60 – 2.37 (m, 3H), 2.27 (dt, *J* = 15.8, 7.3 Hz, 1H), 1.86 (t, *J* = 6.9 Hz, 1H), 1.44 – 1.28 (m, 10H). ^13^C NMR (75 MHz, MeOD) δ 175.0, 173.4, 172.4, 163.4, 162.9, 149.2, 148.6, 146.1, 146.0, 144.8, 129.4, 127.6, 126.5, 126.0, 94.7, 69.2, 66.4, 60.2, 54.4, 47.7 (polyethylene glycol chain), 42.4, 40.1, 39.1, 38.9, 37.7, 34.4, 32.5, 27.7, 26.8, 26.8, 25.5, 25.0, 17.4, 15.9, 11.8.

### **HS-PEG5K-ZW (18)**

Compound **17** (159 mg, 29 μmol, 1.0 equiv) was dissolved in dichloromethane (5 mL), the mixture cooled to 0 ºC using an ice bath, and triisopropylsilane 297 μL, 1.45 mmol, 50.0 equiv) was added, followed by trifluoroacetic acid (222 μL, 2.9 mmol, 100.0 equiv). As soon as the trifluoroacetic acid was added, the clear mixture turned to an intense yellow colour, indicating the formation of the trityl radical. The colour quickly faded, confirming the reduction of the free trityl radical to the colourless triphenylmethane by-product by the silane reagent. The procedure was repeated one more time, then the mixture was allowed to warm to room temperature and the stirring continued for 30 min. The mixture was then concentrated under reduced pressure, the waxy residue triturated twice with cold hexane and sonicated to aid separation from the silane residues, the solvent was carefully removed each time by aspiration with a glass pipette, taking care not to remove the solid material. The white solid was then triturate twice more with cold diethyl ether and dried under high vacuum. The resulting off-white solid was found to produce a slightly cloudy solution when dissolved in halogenated solvents such as chloroform or dichloromethane, thus a solution of the material in 4 ml of dichloromethane was clarified by filtering through a 0.22 μm syringe filter. Concentrating the resulting filtrate under reduced pressure afforded compound **18** as a white fluffy solid (141 mg, 93% yield). The compound was stored in a glass vial flushed with nitrogen prior to sealing and kept at - 20 ºC to minimise dimerisation of the free thiol. ^1^H NMR (300 MHz, MeOD) δ 9.24 (dd, *J* = 9.7, 6.5 Hz, 2H), 8.50 (d, *J* = 6.4 Hz, 2H), 3.65 (s, 228H - polyethylene glycol chain), 2.89 (td, *J* = 6.9, 2.8 Hz, 2H), 2.62 – 2.44 (m,2H), 2.30 (t, *J* = 7.4 Hz, 2H), 1.85 (p, *J* = 6.6 Hz, 2H), 1.44 – 1.25 (m, 2H). ^13^C NMR (75 MHz, MeOD) δ 175.0, 173.4, 162.9, 149.2, 146.1, 126.0, 94.7, 60.2, 54.4, 47.7 (polyethylene glycol chain), 42.4, 40.12, 39.6, 39.0, 38.2, 37.7, 32.5, 26.8, 25.5, 24.9, 19.7, 18.4, 17.3, 15.9, 11.7.

## **1.3. In vitro experiments**

### **Primary cortical neurons culture**

Cortical (CTX) neurons are dissected from 16-day-old embryos (E16) of Wistar rats (Charles River Laboratory). First, the brains are kept in Leibovitz’s L-15 medium (Gibco), and then the cortex is separated and kept in Neurobasal Plus medium (Gibco). Cells are dissociated using a crystal pipette, and the supernatant is centrifuged for 5 min at 500 g. After centrifugation, the pellet is re-suspended in culture media.

The culture media for primary neurons is prepared by supplementing Neurobasal Plus culture media with glutaMAX (1X), B-27 plus supplement (1X), and penicillin/streptomycin (1%), all purchased from Gibco. All substrates and wells where CTX neurons are cultured undergo a three-step coating process. The first step involves incubating polyethylenimine (0.1%, PEI Mw ~25,000, Sigma) in 1X borate buffer for 1 hour, followed by three washes with sterile H_2_O. The second step includes incubating poly-D-lysine (1 mg/mL) in Dulbecco’s phosphate buffered saline (1X) for 1 hour, washing it three times with sterile H_2_O, and leaving it to dry, all purchased from Gibco. The third step involves incubating Geltrex (1%), form Gibco, in culture media for 24 hours, after which the Geltrex is removed and the cells are immediately seeded. The cells are kept in a humidified incubator (37 °C and 5 % CO_2_).

For every experiment, after 4-5 days in vitro (DIV), the medium is partially changed. After 15 DIV, the neurons are functional, and experiments can be performed.

### **SH-SY5Y neuroblastoma cells culture**

SH-SY5Y neuroblastoma cells are grown in DMEM/F-12 culture medium (Gibco), supplemented with fetal bovine serum (10%) and penicillin/streptomycin (1%). These cells are maintained in culture, and passages are performed every 3-4 days to ensure they are ready for experiments. The cells are kept in a humidified incubator (37 °C and 5 % CO_2_), and the medium is replaced regularly to maintain optimal growth conditions.

### **Assessment of AMPAR expression**

To verify endogenous AMPAR subunit expression in the cellular models used in this study, publicly available transcriptomic datasets were analyzed. Gene expression levels for the AMPAR subunits encoded by the **GRIA1**, **GRIA2**, **GRIA3**, and **GRIA4** genes were extracted from publicly available RNA-sequencing datasets corresponding to the human neuroblastoma cell line **SH-SY5Y cultured in DMEM** and rat primary cortical neurons isolated from embryos at E17-E18.

Transcriptomic datasets containing raw read counts for SH-SY5Y cells were obtained from the NCBI Gene Expression Omnibus (GEO) under accession numbers GSE307689 and GSE285224. Corresponding datasets for rat primary cortical neurons were retrieved from the same repository under accession numbers GSE271487 and GSE268844.

Raw read counts from the respective datasets were first filtered to remove genes with zero counts. The remaining data were normalized using the median of ratios method implemented in the DESeq2 R package to account for sequencing depth and RNA composition bias. The resulting normalized counts were log_10_-transformed and used to rank genes according to their expression levels within each dataset. This approach was utilized to estimate the relative expression of AMPAR subunits (GRIA), which were subsequently compared with the expression profiles of other ionotropic glutamate receptor subunits, specifically kainate (GRIK) and NMDA (GRIN) receptors (**Figures S13 and S14**).

Across all analyzed datasets, AMPAR subunits were consistently detected. In both SH-SY5Y cells and rat primary cortical neurons, several **GRIA** genes were expressed at levels **above or close to the median of the transcriptome-wide expression distribution**, indicating that AMPAR subunits are readily expressed in these neuronal models. In particular, **GRIA1**, **GRIA2**, and **GRIA3** showed moderate-to-high expression ranks relative to the full gene set in both datasets analyzed for each cell type.

**Figure S13. AMPAR gene expression in SH-SY5Y cells.** Transcriptome-wide gene expression ranked by normalized counts (log_10_ scale) using RNA-seq datasets **GSE307689** (Dataset 1) and **GSE285224 (Dataset 2)**. Grey dots represent all genes, while colored markers indicate glutamate receptor genes, highlighting AMPAR subunits (**GRIA1**, **GRIA2**, **GRIA3**, **GRIA4**).

**Figure S14. AMPAR gene expression in primary rat cortical neurons.** Transcriptome-wide gene expression ranked by normalized counts (log_10_ scale) using RNA-seq datasets GSE271487 **(Dataset 1)** and GSE268844 **(Dataset 2)**. Grey dots represent all genes, while colored markers indicate glutamate receptor genes, highlighting AMPAR subunits (**GRIA1**, **GRIA2**, **GRIA3**, **GRIA4**).

### **Pharmacological validation of AMPAR functional expression**

Cultures at DIV 42 were used, following the same culture conditions described above. Spontaneous network activity was first recorded by MEA to establish baseline activity. Cultures were then treated with the AMPAR antagonist 6,7-Dinitroquinoxaline-2,3(1H,4H)-dione (DNQX 5 µM, Sigma Aldrich) and activity was recorded immediately after treatment. Following 30 min of incubation with the antagonist, a further recording was taken. The antagonist-containing medium was then removed and replaced (washout), and activity was recorded 30 min and 60 min after washout. MFR was calculated per electrode at each time point and normalized to that electrode's own baseline value, following the same procedure described for the AuBP-targeting experiments (see above). 16 electrodes were analysed for the DNQX condition.

### **Attachment of AuBPs to cell membrane**

CTX neurons or SH-SY5Y cells were exposed to CAM2-TCO linker solutions (5 µM, 200 µL) for 1 hour. Subsequently, the linker solution is removed, and a dispersion of AuBPs@PEG-Tz/Zw (0.02 or 0.002 nM, 200 µL) is added and incubated for 30 min, followed by the removal of unlinked nanoparticles and replenishment of fresh culture media. As control, cells are incubated with either the linker or the BPs. Importantly, the culture medium from each well was consistently used for both linker and AuBPs@PEG-Tz/Zw incubations, thereby avoiding alterations in neuron behaviour due to changes in the culture medium.

### **Confocal imaging**

SH-SY5Y cells were seeded (7x10^4^ cells/well) and grown for 2 days on 8 multi-well Ibidi slides. AuBPs@PEG-Zw/Tz/Alexa488 (0.002 nM) were incubated for 0.5, 1, 3, or 24 h, with and without prior incubation with the linker. After a given time, the media were removed, and the cells were washed gently with DPBS and fixed with paraformaldehyde (4 %) for 20 min, and again washed three times with DPBS. Actin filaments of the cytoskeleton were labelled with Texas red-phalloidin (160 nM) in DPBS for 20 min, and the nuclei with DAPI (300 nM) in DPBS for 5 min. In between 3 washing steps of 5 min are performed with DPBS, all in RT. Images were taken with a 63x oil objective in a LS900 Confocal Microscope from Zeiss. The AuBP-to-nucleus fluorescence ratio was determined by quantifying, in ImageJ, the number of Alexa488-positive dots and the number of nuclei across 8 images per condition. The normalization of fluorescence accounted for differences in cell density between images

### **Transmission electron microscopy**

CTX neurons are seeded (10^5^ cells/well) into 8 multi-well slides from Thermo Scientific Nunc Lab-Tek. After 20 DIV cells were treated with CAM2-TCO linker and AuBPs@PEG-Tz/Zw as described in section 2.6. Cells were fixed with glutaraldehyde (30% in 0.1 M sodium phosphate buffer) for 10 min at 37 °C and then the glutaraldehyde is refreshed and incubated for 1h at RT. Samples were sent to Centro de Investigación Príncipe Felipe (CIPF) for grids preparation. Fixed samples were embedded in Epoxy resin (Agar Scientific) and ultrathin sections (50 nm) were cut with a Leica ultramicrotome for grids preparation at entro de Investigación Príncipe Felipe (CIPF). TEM images were acquired with a FEI Tecnai G2 20 TWIN transmission electron microscope operating at an acceleration voltage of 200 kV. The AuBPs@PEG-Tz/Zw (0.002 nM) were incubated for 1 and 24 hours without or with pre-incubation of CAM2-TCO, and the total number of AuBPs@PEG-Tz/Zw is counted in 8 images per condition. Additionally, it was determined whether the BPs are attached to the membrane or internalized.

### **Inductively Coupled Plasma – Mass Spectrometry**

Experiments were conducted in CTX primary rat neurons, incubated for 0.5 h with AuBPs@PEG-Zw/Tz (0.002 nM), both without and with CAM2-TCO pre-incubation. For control experiments, the amount of Au was measured in the absence of any treatment. Following incubation, the neurons were mechanically detached and frozen at -80 °C in QIAzol (Qiagen). Samples (100 μL) were prepared for Inductively Coupled Plasma – Mass Spectrometry (ICP-MS) analysis by microwave digestion with of nitric acid (HNO₃, 750 μL ) and hydrochloric acid (HCl, 250 μL), following a specific digestion program: 160 °C, 30 bar, ramp 10 min, hold 10 min (80 % power), 190 °C, 30 bar, ramp 5 min, hold 20 min (90 % power), and cooling to 50 °C at 25 bar. Calibration solutions were prepared from CMS2 reference material (Inorganic Ventures), diluted in aqua regia (2 % HNO₃ / 0.5 % HCl) to match the sample composition. An internal standard (50 µg/L indium) was added to the calibrations, samples and blanks to improve linearity and minimize instrumental error. The calibration curve showed a linear range of 1-50 µg/L (R² > 0.999).

ICP-MS analysis was performed using the iCAP-Q ICP-MS (Thermo Scientific) equipped with an ASX-500 autosampler (CETAC Technologies). All samples were measured in triplicate and each condition was duplicated to calculate the mean ± standard error. Gold quantification is performed by monitoring the 197Au isotope, with 115In as the internal standard, using the Qtegra v2.6 software (ThermoFisher).

### **Laser-MEAs setup for photothermal neuromodulation and recording**

For neuronal activity recording a Maestro Edge Multielectrode Array system (MEAs) from Axion Biosystems was used. The same company produces multi-well plates that have printed Au electrodes (Cytoview MEA 24) for electrical activity sensing. To the MEAs equipment, a Coherent OBIS 785LX laser system (785 nm, NIR, beam diameter = 0.7 mm) equipped with an OBIS Heat Sink fan for heat management was coupled (**Figure S17**). The laser was controlled by customized Python script.

### **Temperature monitoring during pulsed laser irradiation**

Thermal imaging experiments were conducted to assess temperature changes at microelectrode arrays in the presence and absence of AuBPs@PEG-Zw/Tz under pulsed laser stimulation. Thermograms were recorded with a FLIR A700 thermal camera, which was positioned to provide time-resolved temperature measurements over both the laser spot (directly at the electrodes) and an adjacent reference region unaffected by direct irradiation. Laser stimulation was performed in Neurobasal Plus medium either unsupplemented or supplemented with AuBPs@PEG-Zw/Tz (0.002 nM), allowing evaluation of eventual nanoparticle-mediated bulk heating. The stimulation protocol consisted of successive trains of 10 laser pulses delivered at each of three pulse durations: 100 ms, 200 ms, and 500 ms at increasing laser power density (0 – 26 W/cm^2^). All pulses were spaced by 1 s interval.

### **Neuronal network activity modulation**

CTX neurons were seeded (10^5^ cells/well) on top of the electrodes, and after 15-25 DIV the activity wass recorded for 5 min. For the recording, the plate was placed in the MEAs and stabilized for 15 min (37 °C and 5 % CO_2_). CAM2-TCO and AuBPs@PEG-Zw/Tz ware incubated with the CTX neurons as described in section 2.6. 100, 200 and 500 millisecond-laser pulses were tested in different wells. A total of 10 pulses were delivered every 3 seconds at an increasing laser power density (LPD) of 5.2, 15.6 and 26 W/cm^2^. A full description of the laser parameters used for each condition, including total laser power, pulse repetition rate, duty cycle, energy density per pulse and total energy delivered per pulse train, is provided in **Table S1**. Pulses were delivered continuously at a fixed 3s interpulse interval throughout the entire stimulation protocol, including the transitions between LPD conditions; no additional recovery period was introduced before increasing the power density. The activity was measured during all the time the experiment was performed and changes in the mean firing rate (MFR) were evaluated for each of the electrodes in each experimental condition, while the LPD of the pulses changes. For the analysis, the four corner electrodes of the array were excluded, as were any electrodes showing no activity (MFR < 4 Hz). To ensure accurate data analysis, the artefact detection and removal process identified the time intervals where artefacts occur, corresponding to the laser ON-OFF transitions, and excludes data from these intervals, extending the exclusion window by an additional ±10 ms around each artefact. The artefact removal process consistently applied to all electrode recordings in subsequent analyses. This precaution ensures that all laser-induced distortions were removed from the dataset, preventing them from affecting the measured neuronal activity. Results are shown as the mean and standard error of all the analysed electrodes per well. The One-way ANOVA statistical analyses were done using OriginPro 2016 software and results were considered statistically significant for a p < 0.05. Neuronal activity data for each condition (pulse duration × LPD) were obtained from a single MEA well, derived from one neuronal preparation, with different laser conditions tested in parallel to avoid confounding preparation-to-preparation variability. Electrodes within each well are therefore reported as a measure of spatial variability of the response across a single neuronal network, rather than as independent biological replicates. Electrodes with low baseline activity (as described above, MFR < 4 Hz over the full recording, or lacking detectable activity in the baseline window used for normalization) were excluded from analysis. The number of electrodes analyzed per condition is reported in **Table S2. To assess whether laser-induced suppression of neuronal activity persisted beyond the stimulation period, MFR was additionally computed for the recording window between the end of the last laser pulse and the end of the recording and compared to each electrode's baseline MFR.**

**Table S1.** Laser stimulation parameters used for photothermal neuromodulation experiments. For each pulse duration and laser power density (LPD) tested, the corresponding total laser power, pulse repetition rate, duty cycle, energy density per pulse, and total energy delivered per 10-pulse train are listed. Beam spot diameter = 0.7 mm (spot area = 3.85 × 10⁻³ cm²); total laser power = LPD × spot area.

| **Pulse duration (ms)** | **Power density during pulse (W/cm²)** | **Total laser power during pulse (mW)** | **Pulse repetition rate (Hz) / Period** | **Duty cycle (%)** | **Energy density per pulse (J/cm²)** | **Energy delivered per pulse (mJ)** | **Total energy delivered per train (10 pulses, mJ)** |
| --- | --- | --- | --- | --- | --- | --- | --- |
| 100 | 5.2 | 20 | 0.33 Hz / 3 s | 3.3 | 0.52 | 2.0 | 20 |
| 100 | 15.6 | 60 | 0.33 Hz / 3 s | 3.3 | 1.56 | 6.0 | 60 |
| 100 | 26.0 | 100 | 0.33 Hz / 3 s | 3.3 | 2.60 | 10.0 | 100 |
| 200 | 5.2 | 20 | 0.33 Hz / 3 s | 6.7 | 1.04 | 4.0 | 40 |
| 200 | 15.6 | 60 | 0.33 Hz / 3 s | 6.7 | 3.12 | 12 | 120 |
| 200 | 26.0 | 100 | 0.33 Hz / 3 s | 6.7 | 5.20 | 20 | 200 |
| 500 | 5.2 | 20 | 0.33 Hz / 3 s | 16.7 | 2.60 | 10 | 100 |
| 500 | 15.6 | 60 | 0.33 Hz / 3 s | 16.7 | 7.80 | 30 | 300 |
| 500 | 26.0 | 100 | 0.33 Hz / 3 s | 16.7 | 13.00 | 50 | 500 |

**Table S2**. Number of electrodes analysed per condition in Figure 4E-G.

| **Pulse duration** | **Condition** | **N electrodes** |
| --- | --- | --- |
| 100 ms | Control | 13 |
| 100 ms | Linker | 13 |
| 100 ms | BPs | 13 |
| 100 ms | Low Linker+BPs | 14 |
| 100 ms | High Linker+BPs | 10 |
| 200 ms | Control | 10 |
| 200 ms | Low Linker+BPs | 10 |
| 500 ms | Control | 10 |
| 500 ms | Low Linker+BPs | 9 |

### **Temperature experiment in MEAs with CTX Neurons**

The temperature experiment was conducted using CTX primary rat neurons cultured in a 24-well plate (10^5^ cells/well). The bulk temperature was increased from 31 °C to 41 °C in 1 °C increments. At each temperature step, the temperature was held for 1 min, and neuronal activity is measured. After reaching 41 °C, the temperature was gradually decreased back to 31 °C, following the same 1 °C steps and measuring activity at each temperature.

For data analysis, only wells that show an average MFR greater than 4 Hz across all 16 electrodes were considered. The average neuronal activity and the standard error of the mean (SEM) are calculated for the wells that meet this criterion. The change in activity, represented as MFR, was then analysed to determine how the neuronal activity is affected by temperature changes.

### **Computational Analysis**

Raw files obtained from the Maestro Edge MEAs were preprocessed using Axion Biosystems MatLab® scripts to extract the raw signal from each electrode in the recordings. The raw signal was subsequently processed using the spikeinterface library (v0.102.1) following these steps: First, raw signal files were loaded, creating a separate object for each well. Next, a bandpass filter within the frequency range of 300–5000 Hz was applied to the signals. To determine the background noise level, trace re-referencing was performed using the common_reference() function with the parameter reference='global', followed by noise level computation using the get_noise_levels() function. Spike detection was then carried out using the detect_peaks() function with the 'locally_exclusive' method, setting a detection threshold of σ=5 based on the previously computed background noise level. To eliminate potential bias, spikes occurring within ±10 ms of laser exposure were removed, restricting subsequent analysis to spikes generated outside of these exposure intervals. Electrodes were excluded from analysis if their mean firing rate across the entire recording fell below 4 Hz (i.e., electrodes with fewer than 4 spikes per second were discarded), as firing rate estimates below this threshold are based on very few detected events and are therefore unreliable, with an increased risk that detected events reflect background noise rather than genuine neuronal activity. Following this criterion, electrodes located at the four corners were removed, along with additional electrodes from specific wells based on activity levels. After filtering, the MFR was calculated using spikes occurring between the first and last repetitions, with an additional extension of 3 s. The normalized MFR was computed across laser powers, calculated as the ratio of the MFR at a specific laser power to the MFR at laser power 0. Lastly, burst frequency was calculated for each well and laser power using the following procedure. Initially, spike counts were summed across electrodes using a time window (tw) of 10 ms, and the resulting sum was normalized by dividing it by the number of active electrodes and tw, yielding spikes per electrode per second. These normalized spike counts were ranked, and the knee point of their distribution was computed using the kneed library (v0.8.5). The determined cutoff was divided by per min (0.10) to define bursts as time intervals [t0, t1], where all normalized spike counts exceeded this cutoff. Burst detection was further refined by merging bursts separated by intervals shorter than 200 ms and discarding bursts lasting less than 100ms.

### **Cytotoxicity and phototoxicity evaluation**

Cytotoxicity induced by linker and BPs and laser treatment was assessed by lactate dehydrogenase (LDH) leakage and neurofilament light chain (NfL) release into the culture medium. Culture media was aspirated before and after the CAM2-TCO and AuBPs@PEG-Zw/Tz are incubated and laser treatment. The activity of LDH in the medium was determined using a commercially available kit from MedChemExpress. The assay is based on the conversion of lactate to pyruvate in the presence of LDH with parallel reduction of NAD. The formation of NADH from the above reaction results in a change in absorbance at 490 nm. Aliquots of media and LDH reagent were mixed in a 96-well plate (Fisher) and absorbance at 490nm is recorded using a microplate spectrophotometer system (GloMax Microplate reader from Promega).

NfL was quantified using the commercial EllaTM microfluidic platmorn (Bio-Techne, Minneapolis, MN, USA) following the kit instructions. Briefly, diluted media (1/2) was incubated in the SimplePlex® rat NfL assay cartridge (#SPCKB-RP-003406). Then, the wash buffer (1 mL) was added to the specific wells, and 50 µL of the high and low concentration of NfL is used as the internal control. The average of the triplicated measurements of NfL concentration obtained for each sample is used in correlation analysis.

### **Statistical analysis**

All the one-way ANOVA statistical analyses are done using OriginPro 2016 software.

# **2. Results and discussion**

### **Comment on the antifouling effect of zwitterion groups**

The hydrodynamic diameter of the gold nanospheres functionalized with PEG-COOH increases by 5.6 nm (from 56.3 to 61.9 nm) when the nanoparticles are incubated in the medium for 24 h, however, when the nanoparticles are functionalized with PEG-Tz/PEG-Zw, the diameter shows a very slight increase (from 54.9 to 55.1 nm, within the statistical margin of error of the measurement) due to the antifouling effect of the Zw groups (Figure S8). We also performed Z-potential measurements in water and in Neurobasal plus medium (Figure S8). Both samples exhibited a decrease in Z-potential. The charge of PEG-COOH-functionalized spheres decreases from -45.9 mV (water) to -20.8 mV (medium), and for PEG-Zw/Tz-functionalized spheres decreases from -23.2 mV (water) to -6.1 mV (medium), being more pronounced for the PEG-COOH-functionalized spheres. In both samples, the value of Z-potential gradually approaches neutral charge due to the electrostatic screening effect caused by ionic forces.

**Figure S15.** Pharmacological validation of functional AMPAR expression in primary cortical neuron cultures. Normalized mean firing rate (MFR, basal = 1) per electrode, recorded by MEA following treatment with the AMPAR antagonist DNQX (5 µM). Recording sequence: baseline (Basal); immediately after antagonist application (Treatment T0); after 30 min of incubation with the antagonist (Treatment T30); 30 min after washout (Washed T30); and 60 min after washout (Washed T60). Each grey point represents one electrode; red lines indicate the mean ± SD across electrodes at each time point. The dashed grey line marks the basal reference (normalized MFR = 1).

### **Comment on pharmacological validation of functional AMPAR in primary CTX neurons**

DNQX application produced a marked decrease in normalized mean firing rate (MFR) at Treatment T0 relative to baseline (**Figure S15**), confirming that AMPARs are functionally expressed and pharmacologically active at the plasma membrane of these cultures. Activity showed partial recovery by Treatment T30, consistent with the binding-unbinding equilibrium kinetics expected for a reversible competitive antagonist rather than a non-specific or toxic effect on the network, and returned toward baseline levels following washout (Washed T30-T60).

Together with the AMPAR transcript-level validation reported in **Figures S13 and S14**, this pharmacological result confirms functional AMPAR expression in the cell preparations used for targeting experiments, and supports the interpretation that the linker-dependent nanoparticle association observed in **Figures 2 and 3** (main text) is consistent with AMPAR-mediated binding.

**Figure S16.** Evaluation of linker-mediated AuBPs membrane association and cellular interaction though confocal imaging. Confocal fluorescence images of SH-SY5Y neuroblastoma cells (actin in orange, nuclei in purple), and after1h and 3 h incubation with AuBPs (green) without (–) and with (+) previous linker incubation.

**Figure S17.** Confocal fluorescence images of SH-SY5Y neuroblastoma cells without treatment (control group). A merged image is shown (actin in orange, nuclei in purple, AuBPs in green) and the green channel to prove the absence of green florescence in the control.

**Table S3.** Quantified number of AuBPs inside the neurons (In) and on the membrane (Out) from TEM images in primary rat CTX neurons. A total of 8 TEM images per condition are analysed to quantify the distribution of AuBPs (0.02 nM) in primary rat CTX neurons after 30 min and 24 h of incubation, comparing no-linker and linker (5 µM) conditions. The analysis differentiates between AuBPs located inside (In) and outside (Out) the cells.

|  | **30min** | | | | | | **24h** | | | | | |
| --- | --- | --- | --- | --- | --- | --- | --- | --- | --- | --- | --- | --- |
|  | **No linker** | | | **Linker** | | | **No linker** | | | **Linker** | | |
| **N** | **Total** | **In** | **Out** | **Total** | **In** | **Out** | **Total** | **In** | **Out** | **Total** | **In** | **Out** |
| **1** | **0** | **0** | **0** | **3** | **0** | **3** | **1** | **0** | **1** | **31** | **30** | **1** |
| **2** | **0** | **0** | **0** | **2** | **0** | **2** | **1** | **1** | **0** | **40** | **40** | **0** |
| **3** | **0** | **0** | **0** | **7** | **0** | **7** | **2** | **2** | **0** | **6** | **0** | **6** |
| **4** | **3** | **0** | **3** | **7** | **0** | **7** | **1** | **1** | **0** | **20** | **19** | **1** |
| **5** | **1** | **1** | **0** | **5** | **0** | **5** | **5** | **5** | **0** | **12** | **0** | **12** |
| **6** | **1** | **1** | **0** | **2** | **0** | **2** | **17** | **17** | **0** | **58** | **55** | **3** |
| **7** | **9** | **9** | **0** | **1** | **0** | **1** | **6** | **6** | **0** | **60** | **60** | **0** |
| **8** | **2** | **0** | **2** | **1** | **0** | **1** | **5** | **5** | **0** | **6** | **0** | **6** |
| ***Total BPs*** | ***16*** | ***11*** | **5** | ***28*** | ***0*** | ***28*** | ***38*** | ***37*** | ***1*** | ***233*** | ***204*** | ***29*** |
| ***Out/In Ratio*** | ***0.45*** | | | ***1*** | | | ***0.03*** | | | ***0.14*** | | |

**Figure S18.** A) Digital image of in-house made system to couple Obis laser (785 nm) to MEAs system for neuromodulation experiments. B) Schematic illustration of the laser lighting the electrodes: Microelectrode array of a single well from the multi-well plate (Axion Biosystems) used in MEA-based neuromodulation experiments. The red circle indicates the 785 nm laser beam, which is centred on the 16-electrode square array. For data analysis in Figures 4 and 5, the four corner electrodes were excluded.

**Figure S19.** Temperature change on electrodes exposed to laser pulses. The laser light of pulse duration from 100 ms, 200 ms to 500 ms was applied to electrode immersed in Neurobasal Plus medium in the absence of bipyramids, AuBPs(–) left column, and in the presence of gold bipyramids, AuBPs(+), 0.002 n, right column. The values of ΔT were obtained by normalizing each trace to its initial temperature, with recordings performed at the laser spot (black) and at a reference region (grey). Red shaded intervals indicate laser ON periods. No increase of bulk temperature was recorded.

**Figure S20.** Artefacts due to laser on-off. 10 pulses of 100ms at an increasing LPD (from 0 to 26 W/cm2) are applied and the artefacts are detected. The applied LPD has an effect on the amplitude of the artefacts detected on the MEAs. These corrections enhance the reliability of the analysis by ensuring that observed neuromodulation effects result from neuronal responses rather than technical artefacts.

**Figure S21.** Identification and removal of laser-induced artefacts in MEAs recordings: this figure shows electrode activity plots demonstrating artefacts caused by the ON-OFF transitions of the laser during neuromodulation experiments. The artefacts appear as signal disturbances, in 2 to 4 electrodes of the electrode matrix, at the moments when the laser turns ON and OFF. Additionally, the amplitude of the background signal changes while the laser remains ON, suggesting an influence of laser exposure on the recorded signal. To ensure accurate data analysis, the artefact detection and removal process identifies the time intervals where artefacts occur, corresponding to the laser ON-OFF transitions, and excludes data from these intervals, extending the exclusion window by an additional ±10ms around each artefact. This is done for all the 16 electrodes of the matrix. This precaution ensures that all laser-induced distortions are removed from the dataset, preventing them from affecting the measured neuronal activity. This figure presents examples for laser pulse durations of 100ms, 200ms and 500ms, illustrating how artefacts appear and how they undergo systematic removal. The artefact removal process consistently applies to all electrode recordings in subsequent analyses.

**Figure S22.** Influence of the bulk temperature changes to the MFR of cultured primary CTX neurons. Measurements are performed in a total of 15 wells, with MFR values normalized to the activity recorded at 37°C. As the temperature rises from 37°C to 41°C results indicate that the average MFR at 38°C drops to approximately 58%, at 39°C to 36%, at 40°C to 23%, and at 41°C to 14%. These results indicate a strong negative correlation between temperature and neuronal activity, with higher temperatures leading to a significant reduction in firing rate.

**Figure S23.** Influence of bulk temperature changes on the spike amplitude of cultured primary CTX neurons. The mean spike amplitude (line) ± SEM (shaded area) is shown across temperatures ranging from 31 to 41 °C (heating phase, red) and back from 41 to 31 °C (cooling phase, blue), averaged over a total of 120 electrodes across 15 wells (8 central electrodes per well). Amplitude decreases progressively above 37 °C during heating, reaching a ~10.2% and ~10.9% reduction at 40 and 41 °C, respectively, relative to the value at 37 °C. Upon passive cooling, the amplitude partially recovers, indicating that the thermal effect on spike amplitude is largely reversible. These results indicate a negative correlation between temperature above 37 °C and neuronal spike amplitude, consistent with thermally driven reductions in extracellular medium resistance and accelerated Na⁺ channel inactivation at elevated temperatures.

**Figure S24. Representative bright-field images of primary cortical neurons following photothermal stimulation.** Bright-field micrographs of CTX primary neuronal cultures on MEA wells, acquired after incubation with the linker and AuBPs@Zw/Tz and subsequent photothermal laser stimulation (500 ms pulses). Images were acquired at the periphery of the electrode area, where individual neurons and neurite processes can be more clearly resolved than within the densely seeded central electrode field (see Materials and Methods). Scale-bar: 50µm.

### **Comment on thermal suppression of neuronal activities**

The thermal suppression of neurons can stem from the combined influence of temperature on membrane conductance, ion channel kinetics and synaptic transmission, which together reduce the probability of sustained action potential generation^[9]^. Specifically, elevated temperature accelerates the activation and inactivation kinetics of voltage-gated Na⁺ channels, enhances delayed-rectifier and leak K⁺ currents, and alters the availability of T-type Ca²⁺ channels, collectively increasing spike threshold, shortening action potential duration and deepening after-hyperpolarization, thereby reducing repetitive firing^[10]^. In parallel, temperature-dependent changes in membrane fluidity and capacitance further influence excitability by modifying channel gating and charge redistribution dynamics. Rapid temperature transients may alter the membrane’s electrical capacitance, producing capacitive currents that shift the resting potential, described as optocapacitance mechanism^[11]^. Increased membrane fluidity can further modulate ion channel open probability and kinetics^[12]^. In addition, thermosensitive channels such as TRP and TREK family members are directly activated by temperature fluctuations, leading to changes in conductance and membrane potential^[13]^.

**Figure S25.** Neuronal activity recordings while 100 ms duration laser pulses application. The raster plots represent the activity of active electrodes during the application of 10 pulses of 100 ms duration (in red) at increasing LPDs (0 5, 16, and 26 W/cm²) for the control condition. Each row corresponds to a different LPD, and each vertical line indicates a spike event in all the analysed electrodes.

**Figure S26.** Neuronal activity recordings while 100 ms duration laser pulses application. The raster plots represent the activity of active electrodes during the application of 10 pulses of 100 ms duration (in red) at increasing LPDs (0, 5, 16, and 26 W/cm²) for neurons treated with linker (5μM, 30min). Each row corresponds to a different LPD, and each vertical line indicates a spike event in all the analysed electrodes.

**Figure S27.** Neuronal activity recordings while 100 ms duration laser pulses application. The raster plots represent the activity of active electrodes during the application of 10 pulses of 100 ms duration (in red) at increasing LPDs (0, 5, 16, and 26 W/cm²) for neurons treated with BPs (0.02 nM, 30min). Each row corresponds to a different LPD, and each vertical line indicates a spike event in all the analysed electrodes.

**Figure S28.** Neuronal activity recordings while 100 ms duration laser pulses application. The raster plots represent the activity of active electrodes during the application of 10 pulses of 100 ms duration (in red) at increasing LPDs (0, 5, 16, and 26 W/cm²) for neurons treated with linker (5μM, 30min) and BPs (0.02nM, 30min). Each row corresponds to a different LPD, and each vertical line indicates a spike event in all the analysed electrodes.

**Figure S29.** Neuronal activity recordings while 100 ms duration laser pulses application to the treated CTX neurons. The raster plots represent the activity of active electrodes during the application of 10 pulses of 100 ms duration (in red) at increasing LPDs (0, 5, and 26 W/cm²) to the CTX neurons (upper plot: Control) and when the neurons are treated with linker (0.5 µM, 30min) and BPs (0.002 nM, 30min) (down). Each row corresponds to a different LPD, and each vertical line indicates a spike event in all the analysed electrodes.

**Figure S30.** Neuronal activity recordings while 200 ms duration laser pulses application to the treated CTX neurons. The raster plots represent the activity of active electrodes during the application of 10 pulses of 200 ms duration (in red) at increasing LPDs (0, 5, and 26 W/cm²) to the CTX neurons (upper plot: Control) and when the neurons are treated with linker (0.5 µM, 30min) and BPs (0.002 nM, 30min) (down plot). Each row corresponds to a different LPD, and each vertical line indicates a spike event in all the analysed electrodes.

**Figure S31.** Neuronal activity recordings while 500 ms duration laser pulses application to the treated CTX neurons. The raster plots represent the activity of active electrodes during the application of 10 pulses of 500 ms duration (in red) at increasing LPDs (0, 5, and 26 W/cm²) to the CTX neurons (left: Control) and when the neurons are treated with 0.5µM linker (0.5 µM, 30min) and BPs (0.002 nM, 30min) (right). Each row corresponds to a different LPD, and each vertical line indicates a spike event in all the analysed electrodes.

**Figure S32.** Photothermal modulation of neuronal activity controls for 100, 200 and 500 ms laser pulses application. Normalized MFR following laser stimulation under varying pulses duration and LPDs in neurons.

**Table S2.** Experimental neuromodulation data used for Figure 4G, including AuBP concentration, pulse duration, laser power density (LPD), energy dose per pulse, and the resulting normalized mean firing rate (MFR ± SEM).

| **AuBPs (nM)** | **Pulse (ms)** | **LPD (W/cm²)** | **Energy (J/cm²)** | **MFR (au)** | **MFR SEM** |
| --- | --- | --- | --- | --- | --- |
| 0.02 | 100 | 5.2 | 0.52 | 0.85 | 0.01 |
| 0.02 | 100 | 15.59 | 1.559 | 0.78 | 0.01 |
| 0.02 | 100 | 25.98 | 2.598 | 0.62 | 0.01 |
| 0.002 | 100 | 5.2 | 0.52 | 0.93 | 0.01 |
| 0.002 | 100 | 25.98 | 2.598 | 0.85 | 0.03 |
| 0.002 | 200 | 5.2 | 1.04 | 0.92 | 0.03 |
| 0.002 | 200 | 25.98 | 5.196 | 0.79 | 0.03 |
| 0.002 | 500 | 5.2 | 2.6 | 0.95 | 0.04 |
| 0.002 | 500 | 25.98 | 12.99 | 0.53 | 0.05 |
| 0 | 100 | 5.2 | 0.52 | 0.93 | 0.01 |
| 0 | 100 | 25.98 | 2.598 | 1.02 | 0.02 |
| 0 | 200 | 5.2 | 1.04 | 1.09 | 0.04 |
| 0 | 200 | 25.98 | 5.196 | 0.98 | 0.02 |
| 0 | 500 | 5.2 | 2.6 | 0.98 | 0.05 |
| 0 | 500 | 25.98 | 12.99 | 0.96 | 0.08 |

**Figure S33.** Photothermal modulation of neuronal activity for 100ms laser pulses application. Normalized spike amplitude following laser stimulation under varying pulses duration and LPDs in neurons treated with linker alone, with AuBPs alone and with linker and AuBPs.

**Figure S34.** Photothermal modulation of neuronal activity for 100, 200 and 500ms laser pulses application. Normalized spike amplitude following laser stimulation under varying pulses duration and LPDs in neurons (left) and neurons treated with linker and AuBPs (right).

**Figure S35.** Burst rate as a function of laser power density (LPD), for the conditions shown in Figure 4E-G, at A) 100 ms, B) 200ms and C) 500ms pulse duration.

**Figure S36.** Continuous neuronal activity for the 100 ms pulse duration. Top: firing-rate histogram (200 ms bins, black). Bottom: raster plot (one row per electrode). Colored vertical marks indicate individual laser pulses at each LPD tested.

**Figure S37.** Continuous neuronal activity for the 200 ms pulse duration conditions. Top: firing-rate histogram (200 ms bins, black). Bottom: raster plot (one row per electrode). Colored vertical marks indicate individual laser pulses at each LPD tested.

**Figure S38.** Continuous neuronal activity for the 500 ms pulse duration. Top: firing-rate histogram (200 ms bins, black). Bottom: raster plot (one row per electrode). Colored vertical marks indicate individual laser pulses at each LPD tested.

**Figure S39.** Neuronal activity (MFR, normalized to each electrode's own baseline) immediately before the laser stimulation and after the strongest laser stimulation condition tested, for each treatment condition (top, neurons treated with linker only, AuBPs only and mixture of both exposed to 100ms pulses) and pulse duration (bottom, untreated neurons and neurons treated with linker + AuBPs exposed to 100, 200 and 500ms laser pulses).

# **References**

1. A. Sánchez-Iglesias, and M. Grzelczak, “Expanding Chemical Space in the Synthesis of Gold Bipyramids,” *Small* *21*, no. 2 (2025): 2407735, https://doi.org/10.1002/smll.202407735.

2. J. G. Mehtala, D. Y. Zemlyanov, J. P. Max, N. Kadasala, S. Zhao, and A. Wei, “Citrate-Stabilized Gold Nanorods,” *Langmuir* *30*, no. 46 (2014): 13727–13730, https://doi.org/10.1021/la5029542.

3. R. García-Álvarez, M. Hadjidemetriou, A. Sánchez-Iglesias, L. M. Liz-Marzán, and K. Kostarelos, “In Vivo Formation of Protein Corona on Gold Nanoparticles. The Effect of Their Size and Shape,” *Nanoscale* *10*, no. 3 (2018): 1256–1264, https://doi.org/10.1039/C7NR08322J.

4. J. Piella, N. G. Bastús, and V. Puntes, “Size-Controlled Synthesis of Sub-10-Nanometer Citrate-Stabilized Gold Nanoparticles and Related Optical Properties.,” *Chemistry of Materials* *28*, no. 4 (2016): 1066–1075, https://doi.org/10.1021/acs.chemmater.5b04406.

5. J. Yang, M. R. Karver, W. Li, S. Sahu, and N. K. Devaraj, “Metal-Catalyzed One-Pot Synthesis of Tetrazines Directly from Aliphatic Nitriles and Hydrazine,” *Angewandte Chemie International Edition* *51*, no. 21 (2012): 5222–5225, https://doi.org/10.1002/anie.201201117.

6. N. K. Devaraj, R. Weissleder, and S. A. Hilderbrand, “Tetrazine-Based Cycloadditions: Application to Pretargeted Live Cell Imaging,” *Bioconjugate Chemistry* *19*, no. 12 (2008): 2297–2299, https://doi.org/10.1021/bc8004446.

7. S. Wakayama, S. Kiyonaka, I. Arai, et al., “Chemical Labelling for Visualizing Native AMPA Receptors in Live Neurons,” *Nature Communications* *8* (2017): 14850, https://doi.org/10.1038/ncomms14850.

8. T. Fiala, J. Wang, M. Dunn, et al., “Chemical Targeting of Voltage Sensitive Dyes to Specific Cells and Molecules in the Brain,” *Journal of the American Chemical Society* *142*, no. 20 (2020): 9285–9301, https://doi.org/10.1021/jacs.0c00861.

9. M. J. Van Hook, “Temperature Effects on Synaptic Transmission and Neuronal Function in the Visual Thalamus,” *PloS One* *15*, no. 4 (2020): e0232451, https://doi.org/10.1371/journal.pone.0232451.

10. B. Hille, “Ion Channels of Excitable Membranes,” *Ion Channels of Excitable Membranes*, Vol. 18, 2001.

11. M. Shapiro, K. Homma, S. Villarreal, C.-P. Richter, and F. Bezanilla, “Infrared Light Excites Cells by Changing Their Electrical Capacitance,” *Nature communications* *3* (2012): 736, https://doi.org/10.1038/ncomms1742.

12. T. Tillman, and M. Cascio, “Effects of Membrane Lipids on Ion Channel Structure and Function,” *Cell biochemistry and biophysics* *38* (2003): 161–90, https://doi.org/10.1385/CBB:38:2:161.

13. J. A. Lamas, L. Rueda-Ruzafa, and S. Herrera-Pérez, “Ion Channels and Thermosensitivity: TRP, TREK, or Both?,” *International Journal of Molecular Sciences* *20*, no. 10 (2019): 2371, https://doi.org/10.3390/ijms20102371.
